# COVID-19 Discourse on Twitter in Four Asian Countries: Case Study of Risk Communication

**DOI:** 10.2196/23272

**Published:** 2021-03-16

**Authors:** Sungkyu Park, Sungwon Han, Jeongwook Kim, Mir Majid Molaie, Hoang Dieu Vu, Karandeep Singh, Jiyoung Han, Wonjae Lee, Meeyoung Cha

**Affiliations:** 1 Data Science Group Institute for Basic Science Daejeon Republic of Korea; 2 Korea Advanced Institute of Science and Technology Daejeon Republic of Korea; 3 Electrical and Electronic Engineering Phenikaa University Hanoi Vietnam

**Keywords:** COVID-19, coronavirus, infodemic, infodemiology, infoveillance, Twitter, topic phase detection, topic modeling, latent Dirichlet allocation, risk communication

## Abstract

**Background:**

COVID-19, caused by SARS-CoV-2, has led to a global pandemic. The World Health Organization has also declared an infodemic (ie, a plethora of information regarding COVID-19 containing both false and accurate information circulated on the internet). Hence, it has become critical to test the veracity of information shared online and analyze the evolution of discussed topics among citizens related to the pandemic.

**Objective:**

This research analyzes the public discourse on COVID-19. It characterizes risk communication patterns in four Asian countries with outbreaks at varying degrees of severity: South Korea, Iran, Vietnam, and India.

**Methods:**

We collected tweets on COVID-19 from four Asian countries in the early phase of the disease outbreak from January to March 2020. The data set was collected by relevant keywords in each language, as suggested by locals. We present a method to automatically extract a time–topic cohesive relationship in an unsupervised fashion based on natural language processing. The extracted topics were evaluated qualitatively based on their semantic meanings.

**Results:**

This research found that each government’s official phases of the epidemic were not well aligned with the degree of public attention represented by the daily tweet counts. Inspired by the issue-attention cycle theory, the presented natural language processing model can identify meaningful transition phases in the discussed topics among citizens. The analysis revealed an inverse relationship between the tweet count and topic diversity.

**Conclusions:**

This paper compares similarities and differences of pandemic-related social media discourse in Asian countries. We observed multiple prominent peaks in the daily tweet counts across all countries, indicating multiple issue-attention cycles. Our analysis identified which topics the public concentrated on; some of these topics were related to misinformation and hate speech. These findings and the ability to quickly identify key topics can empower global efforts to fight against an infodemic during a pandemic.

## Introduction

### Background

The COVID-19 pandemic has affected global health and the economy. The use of social media and the internet to seek and share information about the virus has increased rapidly [1,2], which makes them excellent media to examine for patterns of risk communication during a pandemic. During this time, one could observe how the intentional and unintentional spread of misinformation (here defined as unconfirmed or false information) jeopardized public health on such platforms. Studies have shown that people tend to share misinformation faster and more widely than real information [3-5]. The sheer amount of data and the mixture of accurate and false information leaves people confused over which safety guidelines and health tips to follow. This phenomenon has been called an *infodemic* [6]. Infodemics have become a real threat; misinformation on COVID-19 has shifted from focusing on false preventive measures to antivaccination arguments [7] and vandalism toward telecommunication infrastructures [8].

Analysis of risk communication is critical because it helps better understand how and why people propagate or consume certain information upon a threat to their health, economic, or social well-being. Such analysis helps stakeholders prepare and reach informed conclusions about how their decisions affect individuals’ interests, values, and well-being [9]. In the context of COVID-19, which is our interest, analysis of risk communication can find opportunities to mitigate the propagation of false claims that threaten public safety [10].

Studies have identified online risk communication topics by collectively considering temporal tweet trends by adopting, for instance, a statistical clustering method that scans over time [11,12] or a deep learning–based embedding and clustering method [13]. One limitation of statistical approaches is that inaccurate or incomplete input data can act as noise, resulting in unstable clustering results [14]. Embedding approaches for topic modeling have also required that one specify the time duration (eg, monthly). However, such an arbitrary division hinders finding natural topical transitions and critical risk communication topics. Therefore, flexible time durations are preferable in identifying topical shifts.

This research used the data gathered from social media to understand public discourse on COVID-19. Understanding public concerns will help determine which unproven claims or pieces of misinformation need to be debunked first and will contribute to fighting the disease. Primarily, we aim to identify what people say without gatekeeping. For instance, identifying new misinformation in countries that are experiencing a pandemic at an early stage can buy time to debunk the same piece of misinformation in other countries before it poses a threat to public health [10].

To detect meaningful topical shifts of risk communication, one needs to demarcate temporal phases from the public discourse that reflect prevailing circumstances in the real world. If social media conversations were to change by the epidemic phases announced by local governments, one might use the same phases. However, government announcements do not necessarily match with the public interest. Following the issue-attention cycle theory [15], we leveraged drastic changes in the daily tweet volume to divide COVID-19 public discourse online in finding temporal phases. We extracted topics corresponding to the preset temporal phases based on a natural language processing method.

We used a spatiotemporal approach and considered tweets from different countries to provide more holistic views of risk communication. We present views from four Asian countries. Such a multicountry view was used to explore possible opportunities for joint efforts in managing risk communication. For example, early detection of misinformation can help social media services, social media communicators, journalists, policy makers, and medical professionals fight infodemics worldwide.

We ask the following research questions (RQs):

- RQ1: Do the official epidemic phases announced by governments reflect online interaction patterns?
- RQ2: Can topic phases be demarcated automatically based on a bottom-up approach?
- RQ3: What are the major topics corresponding to each topic phase?
- RQ4: What are the unique traits of the topic trends by country, and what are the distinguishing online communicative characteristics?

By answering these RQs, this study makes four contributions. First, we propose an end-to-end method of extracting risk communication topics in a spatial–temporal fashion with less gatekeeping. Second, we provide a theoretical ground (issue-attention cycle) to the framework and successfully assess its validity by observing multiple prominent peaks in the daily conversation. Third, we demonstrate via a case study of four countries a common risk communication trait. During the peak moments of conversation, users on social media concentrate on a few topics. Finally, we show from the case study which topics were directly linked to misinformation and hateful speech in the studied data.

The gathered data from Twitter and the codes (including language tokenizers and analysis codes) are accessible in Multimedia Appendix 1 and on GitHub [16].

### Related Research

#### Issue-Attention Cycle

The issue-attention cycle model [15] conceptualizes how an issue rises into and fades away from the center of public attention. In the first stage, labeled the *preproblem* stage, an undesirable social condition (eg, the appearance of COVID-19) emerges but does not yet draw much public attention. The second stage, dubbed *alarmed discovery and euphoric enthusiasm*, occurs when a triggering event (eg, the national spike of newly confirmed cases of COVID-19) heightens public awareness of the issue. In the third stage, called *realizing the cost of significant progress*, people begin to recognize the hardship involved in restructuring society, and individuals must sacrifice to solve the problem. This causes a *gradual decline of intense public interest*, the fourth stage. In the final *postproblem* stage, the current issue is replaced by a new issue and moves into a twilight zone of reduced public attention.

Not all issues follow the five stages of the issue-attention cycle [17]. As the cyclical patterns of public attention evolve, a wide array of public discourse has been found across multiple issues of climate change [18], emerging technologies [19,20], and public health risks [21,22]. There are also cultural differences in such discourse patterns. For example, concerning the H1N1 pandemic, South Korean news coverage showed five phases of increasing or decreasing attention. The corresponding US news coverage of the pandemic saw only two phases during the same 7-month time period [23].

Despite these fragmented findings, the issue-attention cycle framework provides insights into how public attention dramatically waxes and wanes. An issue that has gone through the cycle is different from issues that have not gone through the cycle in at least two ways. First, when an issue has achieved national prominence, new institutions, programs, and measures will have been developed to address the situation. These developments and their societal impacts are likely to persist even after public attention has shifted elsewhere. Second, the prolonged impacts of these developments are shaped by what was heavily discussed when the issue was of primary public concern.

Although the issue-attention cycle was initially proposed to model traditional media such as newspapers and television, there is a burgeoning literature applying the model to social media platforms. Among them, Twitter serves as a forum that the public is increasingly turning toward to seek and share information that is not subjected to a gatekeeping process [24]. It has become common for journalists to refer to tweets in their news stories. Research has also found that Twitter takes the lead in and exerts control over public discourse, particularly in the early stages of an issue-attention cycle [20,25].

Building on these prior studies, we analyzed Twitter conversations about COVID-19 to examine social media’s issue-attention cycle. We present how to build an end-to-end method of identifying meaningful *topic phases* dynamically. This allows us to compare how issue-attention cycles appear in different countries on the same catastrophic event. To the best of our knowledge, no study has applied dynamic topic modeling in the context of risk communication.

#### COVID-19–Related Analyses

Studies have examined various impacts of the pandemic. Researchers have focused on predicting the transmissibility of the virus. One study estimated the viral reproduction number (R_0_) of SARS-CoV-2, which is known to be more substantial than that of severe acute respiratory syndrome (SARS)–related coronavirus, which was the cause of the SARS outbreak that first appeared in Guangdong Province in southern China in 2002 [26]. Another work based on a stochastic mathematical prediction model of infection dynamics claimed that, by reducing worldwide travel by 90%, the epidemic’s spread could be significantly reduced [27].

Other studies have sought to understand the propagation of misinformation related to COVID-19. One study used an epidemic model to represent the spread of misinformation about COVID-19 on various social media platforms such as Twitter, Instagram, YouTube, Reddit, and Gab; the study showed that users interact and consume information differently on each platform [28]. In this regard, media platforms such as Facebook, YouTube, and Twitter claim to attempt to redirect people to reliable sources of medical information and, to this end, have established direct lines of communication with the Centers for Disease Control and Prevention and the World Health Organization [29].

Among the regional research, one article argued that fake online news in Japan has led to xenophobia toward patients and Chinese visitors [30]. Another study surveyed 300,000 online panel members in South Korea in 2015, when the Middle East respiratory syndrome outbreak was prevalent in this country [31]. This work found that, if public health officials’ information is untrustworthy, people rely more on online news outlets and communicate more via social media.

More recently, a report showed that the public could not easily receive the information on COVID-19 shared by public health officials due to prevalent misinformation on fake cures and conspiracy theories [32]. This study showed that infodemics’ harm varied from country to country depending on public confidence in authorities. One study compared trends in three countries (ie, the United States, the United Kingdom, and Canada) in terms of political bias and found that, although political polarization surrounding COVID-19 exists in the United States and Canada, individuals’ exact perspective on the pandemic is broadly related to the quality of their reasoning skills, regardless of political ideology [33].

Several studies have used data gathered from Twitter to analyze risk communication amid COVID-19. Some of them focus on sentiment analysis based on conventional rule-based lexicon models [34] or deep learning classifiers [35]. These studies measured the degree of sentiment polarity, such as positive and negative, and provided insights from observing daily sentiment changes.

Many types of data sets have been released to the public and research communities on COVID-19. One study crawled Twitter for approximately 3 months and collected information on tweets with relevant keywords in 10 languages [36]. Another work collated over 59,000 academic articles, including over 47,000 research papers, on COVID-19, SARS-CoV-2, and coronavirus-related issues [37] to conduct a comparison study.

#### Topic Modeling–Based Natural Language Processing

Natural language processing such as topic modeling is increasingly used to process extensive documents and extract hidden thematic patterns of textual information [38]. Many studies have explored the capability of topic modeling in understanding the most important subjects of discussion on social media during crises and global epidemics such as dengue [39], Sika virus [40], and Ebola virus [41]. Given the remarkable performance of topic modeling in previous investigations, recent studies on the COVID-19 outbreak have also applied topic modeling to documents collected from different social media sites such as Facebook [42], Weibo [43], and Twitter [44].

One work analyzed COVID-19–related tweets over 2 weeks to study ongoing topics and found that Twitter can be considered a rich medium to understand public opinions in real time [45]. Another work conducted topic modeling on tweets to discover daily hot topics on the pandemic [46]. Furthermore, scholars leveraged Twitter to study the ecosystems of misinformation and conspiracy. One study has shown that users’ political orientation correlates to their contribution to the spread of pandemic-related conspiracy [47]; another study demonstrates the link between fake news exposure and low trust in media [48]. Several techniques have been developed to detect conspiracy and misinformation on social media [49-51].

Despite the growing literature on risk communication during COVID-19, most studies that use topic modeling extract topics from either the entire studied period or manually segmented periods. This study considers time and topics jointly; we used an algorithmic approach to identify topical phases that arise naturally. Our goal is to observe changing risk communication contexts (even when conversations contain similar keywords) from the issue-attention cycle perspective. We also chose to study risk communication in Asian countries that have received relatively little attention. Our data method is not restricted to the studied countries; it can be applied to other languages and countries.

## Methods

### Data

We crawled Twitter for messages by using the Twint Python library [52] and search application programming interfaces [53]. Our analyses focused on four Asian countries (ie, South Korea, Iran, Vietnam, and India). We can ignore possible cultural differences in social media behaviors between Western and Asian users [54,55]. A common platform, Twitter, was used to study public conversations in these countries. Although multiple platforms exist, the open data access and global popularity make Twitter an appropriate medium to conduct a cross-national study.

The four countries were selected as a case study to demonstrate differences in their COVID-19 developments. In Iran, confirmed cases have gradually increased. In contrast, the case count in Vietnam has consistently stayed low. There was an abrupt increase in the numbers after the first confirmed case in South Korea, but the rising curve of confirmed cases has since flattened, unlike other countries. In India, the situation was relatively mild until mid-March 2020, and since then, there has been a drastic surge. Future research can replicate our methodology in other countries.

We set up two keywords, *corona* and *Wuhan pneumonia*, to crawl tweets and collected tweets for the 3 months from January to March 2020. In the studied countries, any tweets containing the official term *COVID-19* in local languages will be searched with the word *corona* (eg, in Korean, it is called *corona-19*). We also added *Wuhan* to collect unofficial terms of the virus. Table 1 lists the keywords used to collect data for each country. Keywords were decided after interviewing multiple local Twitter users for each country.

**Table 1:** Statistics of the scraped tweets.

Language	Duration	Keywords^a^ used	Tweets, n
Korean	January 1 to March 27, 2020	corona, Wuhan pneumonia	1,447,489
Farsi	January 1 to March 30, 2020	#corona, #coronavirus, #Wuhan, #pneumonia	459,610
Vietnamese	January 1 to March 31, 2020	corona, n-CoV, COVID, acute pneumonia	87,763
Hindi	January 1 to March 27, 2020	corona, Wuhan pneumonia	1,373,333

^a^Keywords were used to collect relevant data for each country. We used two kinds of keywords: one official naming of COVID-19 and *Wuhan* as an unofficial representative naming of the virus. Keywords listed here are translated in English from the actual local language (eg, “코로나” means “Corona” in Korean). The original keywords in local languages are listed in Table MA1 in Multimedia Appendix 1.

### Demarcating Topic Phases and Extracting Topics

**Figure 1:**
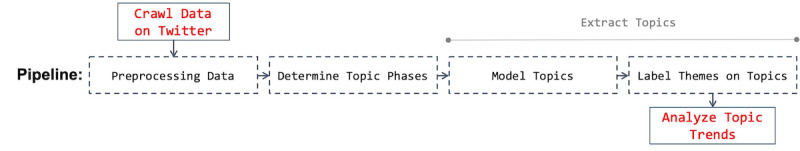
The pipeline structure of the topic analysis.

As shown in Figure 1, the data collection step was followed by the four modules for the extraction and labeling of major topics for certain phases. These steps were repeated for all four countries.

#### Step 1: Preprocessing Data

We first tokenized the data, a process that can be defined as converting data to the smallest units that have meaning. We filtered unnecessary textual information such as stop words, special characters (nonletters), special commands, and emojis. We then used existing Python tokenizer libraries corresponding to each language. Detailed information about the language-specific tokenizers is explained on GitHub [16].

#### Step 2: Determine Topic Phases

The next step is to demarcate specific phases divided by dates to extract topics. This is nontrivial since there are multiple fluctuations and changes in topics reflecting real events such as increased patients with COVID-19. Furthermore, we ruled out using the epidemic phases announced by each government because the offline epidemic phases do not seem to capture actual online topic trends as explained in the forthcoming Basic Daily Trends section.

The issue-attention cycle moderating public attention to a given issue can be measured in media attention, such as the number of news stories [17-19,21-23] or tweets [20,25] on the topic. We, therefore, isolated dates that show sudden increases in the daily tweet volume. We set up two learnable parameters of the first derivatives (hereafter *velocity*) and the second derivatives (hereafter *acceleration*) of the daily tweet volumes, as illustrated in the following equations, where *D* is a day, *t* is a target date, and *t – 1* is 1 day past *t*:


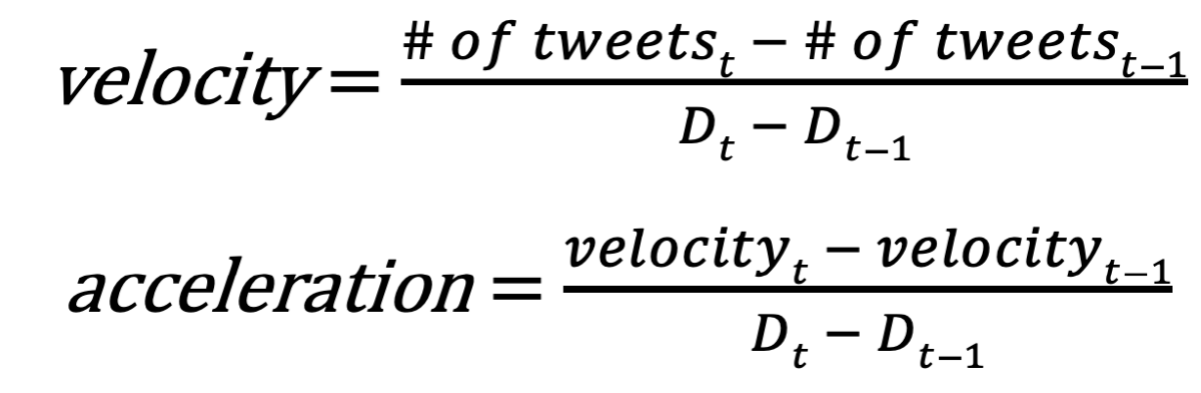


We set the *velocity* and *acceleration* values when *the country announced the first confirmed case* as the ground truth (GT). This approach’s intuition is that *velocity* and *acceleration* are proxies for each country’s unique communication traits regarding a specific subject (ie, COVID-19 in our case). Once these values were computed from the first confirmed date, they were set identically for the remaining periods.

We established joint thresholds for *velocity* and *acceleration* to find dates where *velocity* is still smaller than *velocity_GT_*, and *acceleration* becomes more substantial than *acceleration_GT_* (0<*velocity*<*velocity_GT_* and *acceleration*>*acceleration_GT_*). In this manner, we identified the two parameters from the date of the first confirmed case by country and then detected other dates conjectured to be the start of forthcoming topic phases. When learning these parameters, for *velocity*, we rounded down the *velocity_GT_* value and added 1, and for *acceleration*, we rounded down *accelerationGT*, which is similar to the machine learning approach’s concept of loss minimization (ie, a learning process is finished by one step).

We adopted a low-pass filter with 0.2 as the low-frequency threshold to remove noisy signals and smooth the data. Finally, the temporal data are divided into topic phases (see Multimedia Appendix 1 to find the computed daily *velocity* and *acceleration* trends and demarcated phases by country).

#### Step 3: Extract Topics—Model Topics

We used latent Dirichlet allocation (LDA) for the topic modeling task. LDA is a well-known machine learning method to extract topics from given textual documents (ie, a collection of discrete data points) [56]. LDA generates and maximizes the joint probability of the topics’ word distribution and the documents’ topic distribution [38]. For short sentences, LDA-based methods may not work correctly due to sparse co-occurrences. However, in our case, tweets were collected via specific keywords; therefore, the topics were the focus, and the word co-occurrences among tweets were no longer sparse. Various studies have used the same LDA method on short documents, including Twitter [57-59].

The topic count for each phase is a hyperparameter. The topic count’s range is between 2 and 50. We calculated perplexity, that is, the probability of how many tokens might be placed in the next step (ie, indicating ambiguity over the next possible token). Perplexity is a metric that is often used to optimize language models [60]. The minimum required frequency of words for each phase in tweets was set to 20, and each phase’s epoch (ie, a number of iterations to train LDA) was set to 100. We then decided the optimum number of topics for each phase by choosing the minimum perplexity value. We further analyzed our modeling results’ reliability and confirmed that the results were steady and dependable (see Multimedia Appendix 2 for more details).

Table 2 shows how many prominent topical phases were found for each country. For each phase, we list the statistics of the risk communication, including the period of the topical phase, the total tweet count during the phase, the average user count per day, the average original and retweet counts per day, the ratio of original tweets to retweets, and the number of topics suggested by perplexity.

**Table 2:** The optimal number of phases and topics by country.

Country		Phase 0	Phase 1	Phase 2		Phase 3		Phase 4			Phase 5		
**South Korea**										N/A^a^			N/A
	Time period	Jan 1-19, 2020	Jan 20-Feb 12, 2020	Feb 13-Mar 9, 2020		Mar 10-27, 2020							
	Total tweets, n	507	161,790	672,080		366,073							
	Average users per day	14.06	2415.52	5376.77		5577.88							
	Average original tweets per day	28.17	5244.09	17,796.08		13,095.65							
	Average retweets per day	21.78	56,809.78	211,310.89		147,759.41							
	Tweet depth^b^	0.77	10.83	11.87		11.28							
	Topics determined by perplexity, n	2	41	15		43							
	75th percentile of topics^c^, n	1	18	6		14							
	Final topics^d^, n	1	8	5		11							
**Iran**					N/A		N/A		N/A			N/A	
	Time period	Jan 1-Feb 18, 2020	Feb 19-Mar 30, 2020										
	Total tweets, n	15,473	437,176										
	Average users per day	245.34	1442.46										
	Average original tweets per day	385.63	5272.04										
	Average retweets per day	1315.13	22,128.76										
	Tweet depth	3.41	4.20										
	Topics determined by perplexity, n	3	5										
	75th percentile of topics, n	2	4										
	Final topics, n	3	6										
**Vietnam**													
	Time period	Jan 1-20, 2020	Jan 21-25, 2020	Jan 26-Feb 15, 2020		Feb 16-Mar 4, 2020		Mar 5-22, 2020			Mar 23-31, 2020		
	Total tweets, n	140	1499	18,424		28,458		26,950			12,292		
	Average users per day	3.79	131.25	179.65		485.59		340.65			433.29		
	Average original tweets per day	7.37	218.50	686.60		1238.77		1089.94			1224.00		
	Average retweets per day	0.21	20.75	159.80		582.29		192.24			201.86		
	Tweet depth	0.03	0.09	0.23		0.47		0.18			0.16		
	Topics determined by perplexity, n	19	3	6		46		48			16		
	75th percentile of topics, n	1	1	3		22		19			4		
	Final topics, n	1	2	4		7		10			2		
**India**							N/A		N/A			N/A	
	Time period	Jan 1-29, 2020	Jan 30-Mar 9, 2020	Mar 10-27, 2020									
	Total tweets, n	3088	151,210	1,219,030									
	Average users per day	107.41	1364.95	13,318.63									
	Average original tweets per day	269.72	4261.13	58,924.55									
	Average retweets per day	415.69	14,467.8	318,368.05									
	Tweet depth	1.54	3.40	5.40									
	Topics determined by perplexity, n	3	50	47									
	75th percentile of topics, n	2	22	20									
	Final topics, n	3	5	9									

^a^N/A: not applicable.

^b^Measured as the ratio of retweets to original tweets.

^c^Major topics.

^d^After human annotators merged similar themes.

#### Step 4: Extract Topics—Label Topics

This step involves labeling the themes of the extracted topics and allocating semantic meanings to each topic. We first sorted all tweets with the identified topics in descending order (ie, tweets on the most prevalent topics listed first) and discarded the minor topics that accounted for less than 25% of all tweets.

We then extracted the top 1000 retweeted tweets and the 30 keywords with the highest probability of usage for each topic. We provided these data sets to local users from each country and asked them to label themes for each topic based on the given data sets. Any similar or hierarchical topics were then merged via qualitative coding into a higher category. If one topic corresponded to several themes, then it was given multiple class labels. The maximum number of multiple classes within topics was two, and each class within a topic was weighted as 0.5 in the plot of daily trends in the number of tweets.

Human annotators, who are familiar with the local language and Twitter, qualitatively assessed the extracted topics. First was the intralevel, where annotators labeled each topic based on the contents of the sampled top 1000 tweets and top 30 words. The second was the interlevel, where the annotators compared tweet contents and top-occurring words among topics regardless of the phase. Other annotators then cross-checked the assessment.

The Cohen kappa coefficient to measure the intercoder reliability was 0.766 (see Multimedia Appendix 2 for details on this validation and the list of topics and top-occurring words for each country). Our analysis objective was not to substitute human laborers on monitoring misinformation but to assist them by grouping tweets into specific topics, including misinformation.

Concerning the local and global news themes, we narrowed down the labels since people talked about different news categories. We sublabeled tweets as “_confirmed” if it was about confirmed cases or deaths, “_hate” if it was about hate crimes toward individual races, “_economy” if it was about the economic situation and economic policies, “_cheerup” if it was about supporting each other, and “_education” if it was about when to reopen schools; finally, no sublabel was given to tweets about general information.

## Results

### Basic Daily Trends

**Figure 2:**
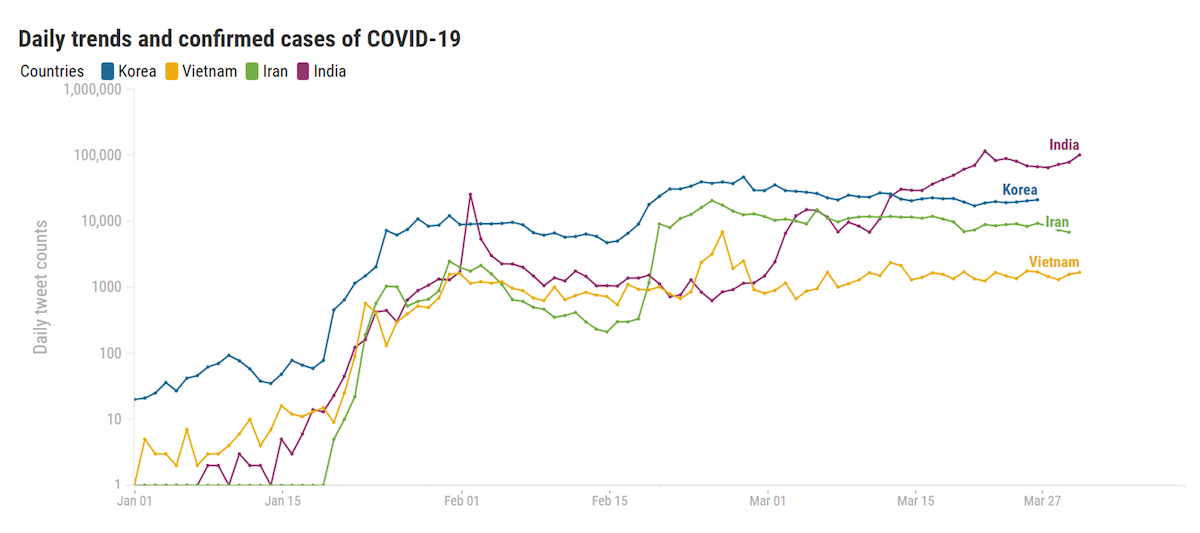
Daily trends in the four countries. The x-axis is dated, and the y-axis is the number of tweets with a log scale.

**Figure 3:**
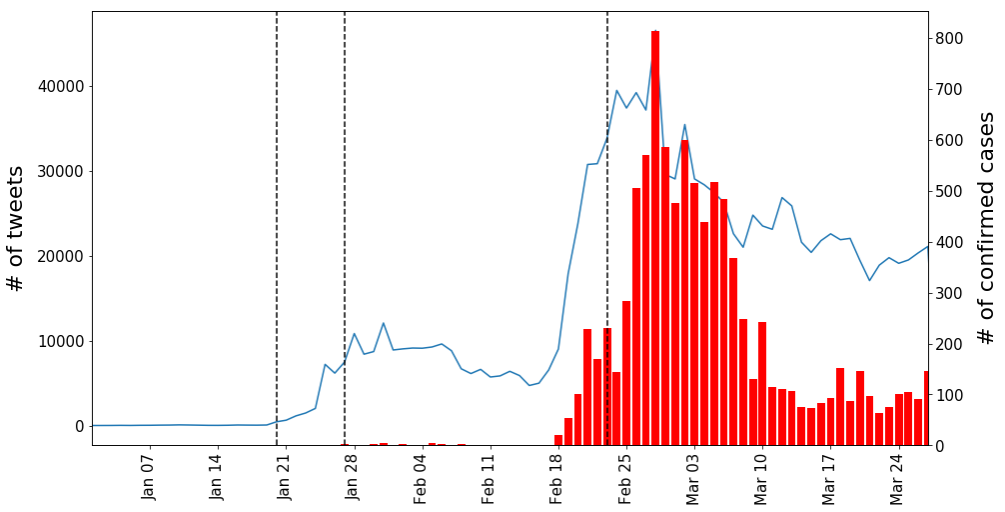
Daily trends in South Korea. Start/end dates of the official epidemic phases (vertical dashed lines), trends in the number of tweets (blue lines), and trends in the number of confirmed cases (red bars).

**Figure 4:**
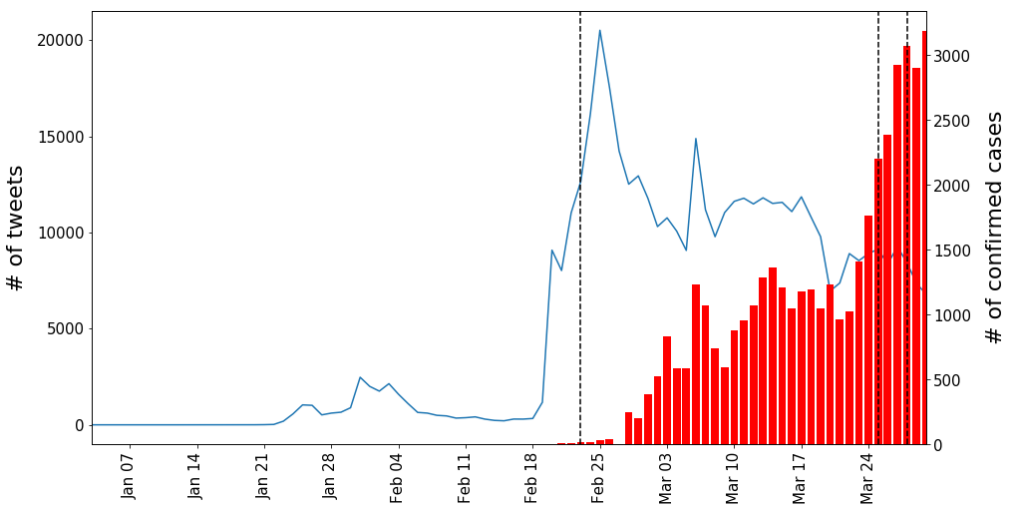
Daily trends in Iran. Start/end dates of the official epidemic phases (vertical dashed lines), trends in the number of tweets (blue lines), and trends in the number of confirmed cases (red bars).

**Figure 5:**
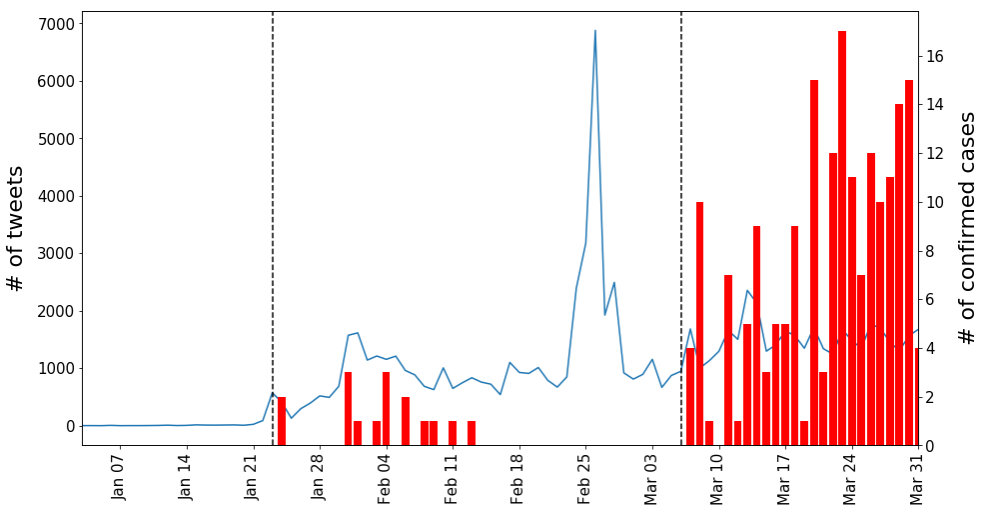
Daily trends in Vietnam. Start/end dates of the official epidemic phases (vertical dashed lines), trends in the number of tweets (blue lines), and trends in the number of confirmed cases (red bars).

**Figure 6:**
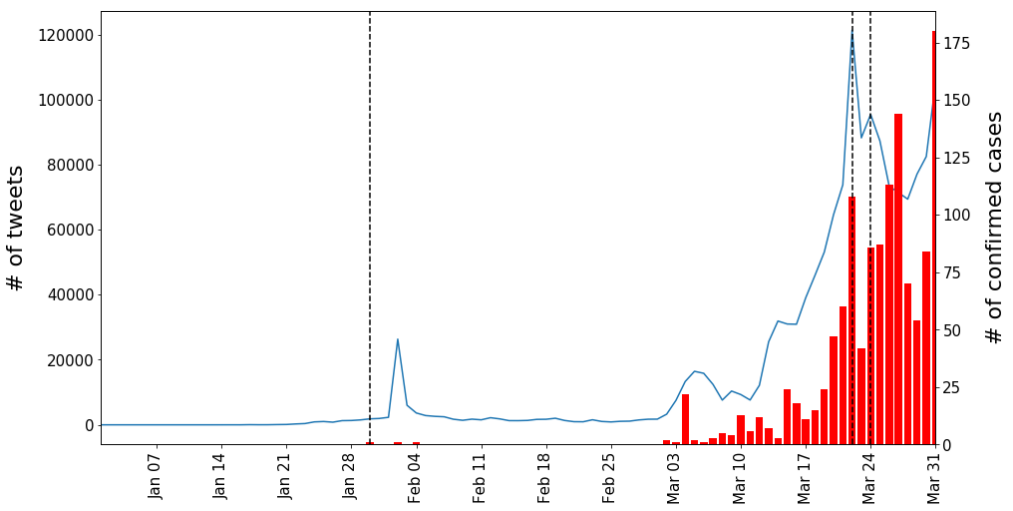
Daily trends in India. Start/end dates of the official epidemic phases (vertical dashed lines), trends in the number of tweets (blue lines), and trends in the number of confirmed cases (red bars).

Figure 2 shows trends of the daily tweet count; the same trend is shown along with the number of confirmed COVID-19 cases for each country in Figures 3-6. Adding to the two trends, we included each government’s official epidemic phases as vertical lines. It is evident in the figures that the tweet trends are associated with the confirmed cases. However, the official epidemic phases do not accurately explain the tweet trends. We examine trends for each country in the following sections.

#### South Korea

The first patient with COVID-19 was reported in South Korea on January 20, 2020. This explains why the tweet count remains relatively low during early January and mostly increases only after late January (see Figure 3). On January 25, the Korean government issued a travel warning for Wuhan and Hubei Province, and suggested that Korean citizens evacuate from those areas, which was heavily discussed on Twitter.

On February 18, 2020, the tweet numbers increased sharply due to the 31st confirmed case related to a cult religious group Shincheonji in Daegu City. After this case was confirmed, the quarantine authority began rigorous testing, focusing on Daegu, and the number of confirmed cases increased drastically until mid-March. The tweet trends follow an identical pattern. However, the official epidemic phases announced by the government, represented by vertical dashed lines in the figure, seem to lag behind the increases in the number of tweets. This pattern shows that the official epidemic phases do not align well with the amount of online attention.

#### Other Countries

We repeated the analysis with the other three countries, as shown in Figures 4-6 (see Multimedia Appendix 3 for each country’s detailed explanation).

### Extracted Topic Trends

We used the daily theme labels acquired from the “Label Topics” module and analyzed the topic changes over time with plots for the four countries. One plot showed daily trends based on the number of tweets, while another plot shows trends based on the number of tweets mentioning country names such as the United States. Overall, as people talked more about the COVID-19 outbreak (ie, as the daily number of tweets increased), people’s topics became less diverse.

#### South Korea

**Figure 7:**
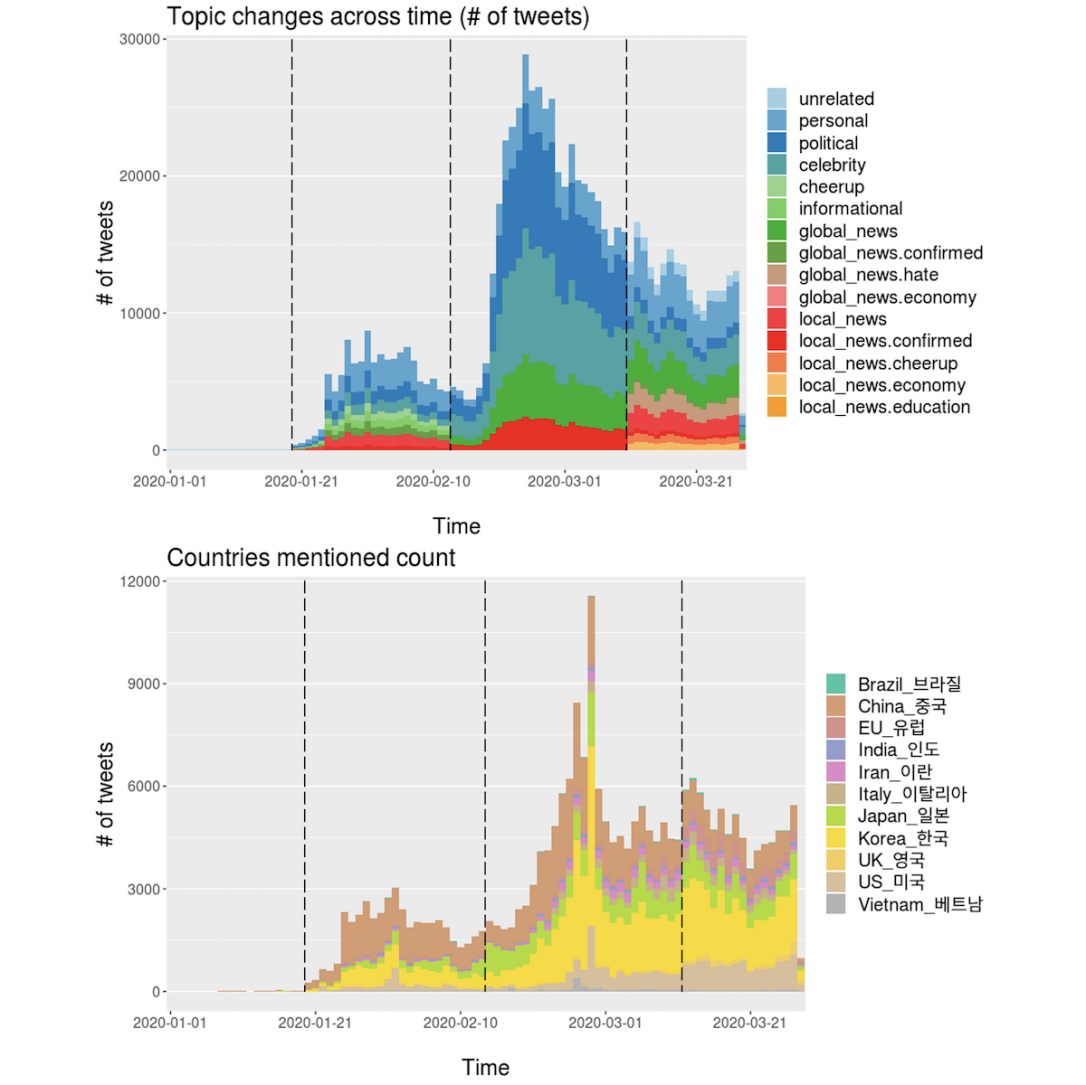
Daily topic trends in South Korea. Trends based on number of tweets (top) and based on number of tweets mentioning country names (bottom).

The data yielded a total of four topic phases, which are used in Figure 7. Phase 0 has no related topics. For phases 1, 2, and 3, the number of topics varies from 8, 5, and 11, respectively. In phase 1, people talked a great deal about their personal thoughts and opinions linked to the current outbreak, and they tried to cheer each other up. In phase 2, as the crisis peaked, people talked less about personal issues and mainly about political and celebrity issues. In Korea, political discussions revolved around closing the South Korean border with China and other countries. In phase 3, as the daily number of tweets decreased relative to that in phase 2, people talked about diverse topics, including local and global news. The major topics here included worries about hate crimes directed toward Asians in Western countries. Such diverse topics are likely shown when people think the pandemic has just passed its peak.

We portrayed daily trends of interest in other countries by counting the tweets mentioning other countries’ names in local languages or English. Korea, China, and Japan were mentioned most frequently; we suspect that this was mainly triggered by political and diplomatic relationships. Meanwhile, the United States and Italy were both mentioned steadily across the 3 months, with the media outlets broadcasting global news affecting this phenomenon.

#### Other Countries

**Figure 8:**
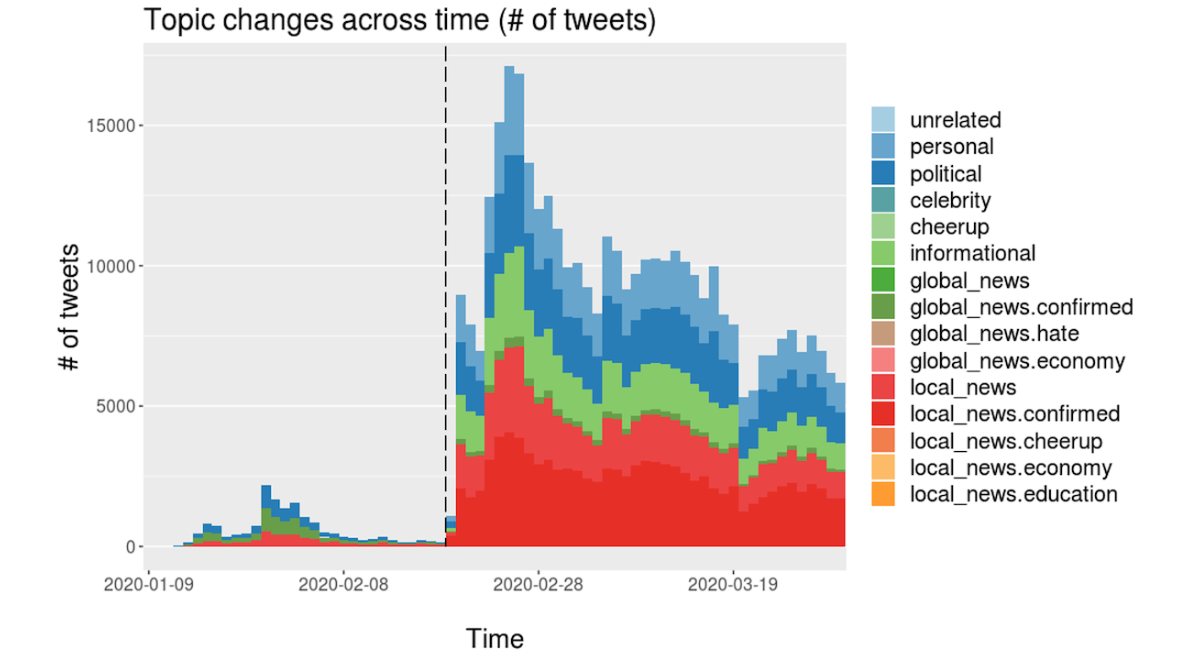
Daily topic trends in Iran based on the number of tweets.

**Figure 9:**
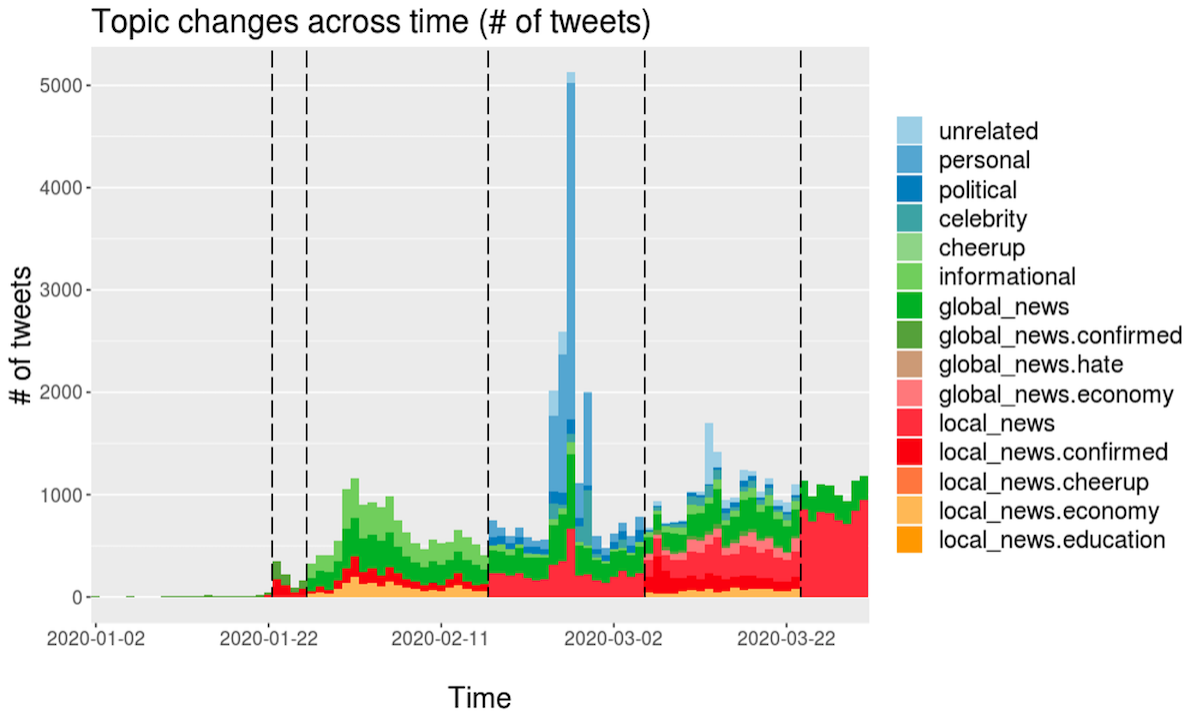
Daily topic trends in Vietnam based on the number of tweets.

**Figure 10:**
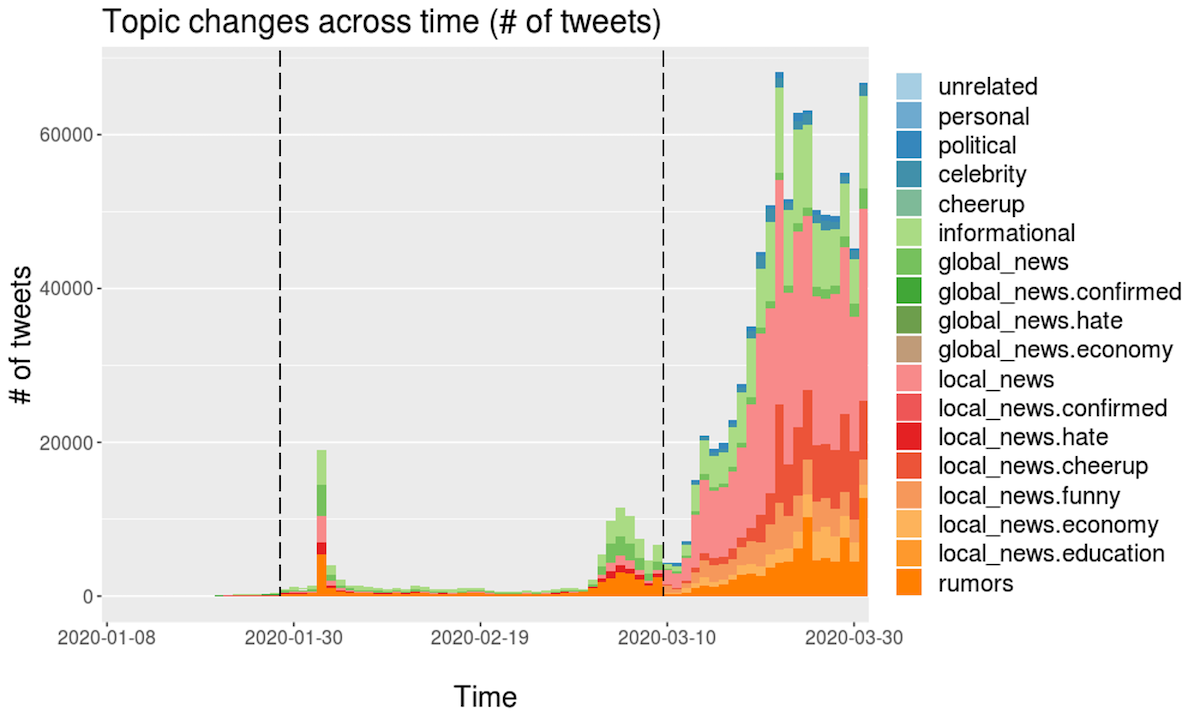
Daily topic trends in India based on the number of tweets.

We repeated the same analysis and interpreted the results for the other cases (Iran, Vietnam, and India), as depicted in Figures 8-10 (see Multimedia Appendix 2 for the derived topic trend and the mentioned country name trend graphs and detailed corresponding explanations by country).

## Discussion

### RQ 1 and RQ 2: Explore an Automatic Way to Decide Topic Phase and Model Topics

This paper analyzes tweets to understand the public discourse on the COVID-19 pandemic. In South Korea, the daily numbers of tweets reached their local maxima in tandem with major offline events. However, in Iran and Vietnam, the tweet counts did not synchronize well with offline events; this may be because of various reasons (eg, Twitter is only one of the platforms used by citizens of this country). Overall, it is interesting to observe that the Twitter data peaks do not necessarily correlate with local governments’ announcements. Social media attention can precede the official announcements, while the official announcements can reinforce the attention.

### RQ 3: Explore Common Traits Among Countries on Risk Communication

Based on the topics labeled as people talked more about COVID-19, they tended to refer to a smaller number of topics. This was more apparent when the tweet depth value was used for the phases, as presented in Table 2.

Tweet depth is defined as the number of retweets per day divided by the number of tweets per day. It can be deemed a measure of standardized cascading depth, with a higher value signifying a greater depth for one tweet. The country-level sociopolitical and cultural background, and Twitter popularity may lead to the observed differences in tweet depth. We verified that tweet depth tended to increase in South Korea and Vietnam cases when people communicated more about COVID-19. This phenomenon reaffirms the finding in another study that the online coronavirus network’s diameter value was smaller than that of other keyword networks [61].

The topical phases with the most considerable tweet depth appeared in the second stage of the issue-attention cycle, where public awareness of an issue soars. In Iran and India, the number of phases might have been too small to discern any such trends. It is also worth noting that this pattern has no intercountry temporal dependence. In other words, even though the pandemic hit the countries at different times, our analysis shows that the tweet depth reached a maximum when the pandemic worsened in that country. This observation could prove to be an effective forewarning of upcoming misinformation cascades.

Moreover, the daily tweet volume peaks reflected the daily number of confirmed cases. In Iran, Vietnam, and India, the daily tweet volume peak anticipated the peak of the number of daily confirmed cases by up to a few weeks. Although the two peaks are close to each other for South Korea, it is worth noting that, around the time of their occurrence, South Korea was becoming the country most affected by COVID-19 outside mainland China.

Interestingly, as shown in Figures 3-6, a simultaneous upsurge in the numbers of tweets occurred in South Korea, Iran, and Vietnam (but not in India) at the end of February 2020, before the upsurge in numbers of locally confirmed cases. Given that COVID-19 is a global issue, this suggests that the issue-attention cycle on a social media platform is more responsive to global rather than local events. In this light, the COVID-19 pandemic offers a gripping opportunity for future researchers to theorize the issue-attention cycle model on a global scale and see how the cycle evolves in conjunction with location-specific topics such as increasing or decreasing numbers of confirmed cases, government measures, and social conflicts.

### RQ 4: Explore Unique Traits by Countries on Risk Communication

We also observed a number of countrywise differences. One of them is the national versus international focus of South Korea and Vietnam during the initial phase. Phase 0 tweets in Korea were not directly related to COVID-19 but simply contained the word *corona* in a different context. This is because this time period was before the first public announcement of the confirmed patients in Korea. In contrast, in Vietnam, the first phase tweets were concerned with international updates on COVID-19. The difference is likely explained by the increasing patient count worldwide. Note that South Korea was one of the first countries to experience the pandemic. We did not attempt to draw any general conclusions from these findings due to the small tweet volumes in phase 0 for both countries. Nevertheless, Vietnamese users discussed the global epidemic more than Korean users from the outset. This tendency may have been associated with Vietnam’s successful defense against the pandemic later on.

With specific reference to each country, in South Korea, when the local (offline) pandemic situation became severe (phase 2), the number of topics discussed on Twitter decreased, which means that people focused more on only a handful of issues. A unique feature of phase 0 was that people sought to cheer each other up and express solidarity in difficult times. In Iran’s case, the topic count was relatively steady over time. The significant topics discussed were confined to news and information; we interpreted this as a sign that Iranian users tend to be cautious about using social media.

For Vietnam, in phase 4, when tweet traffic was lower than in phase 3, the number of topics became more substantial, and the topic themes became less related to the numbers of confirmed cases and death tolls. For instance, people talked more about the economy in phases 2 and 4. The Indian case also displayed a unique trait: many topics were related to misinformation, the scale of which was much lower in the other countries. A large portion of the topics consisted of misinformation and hateful content; this trend was observed throughout phases 2 and 3 (see Multimedia Appendix 2).

### Limitations and Future Work

There are several limitations to be considered. First, we analyzed tweets from only four countries, and therefore, we need to be cautious about extrapolating explanations and insights generally. We plan to extend this study by including more countries. Second, there are other ways to demarcate the topic phases. Our approach was informed by the issue-attention cycle framework, as we computed unique communication traits (ie, *velocity* and *acceleration* by country) that should be relatively consistent across nations throughout the COVID-19 pandemic.

Last, there are also other methodologies to model topics. One natural extension would be to use the external web links that are embedded in the relevant tweets. Scraping the content from external web pages could provide richer contexts in understanding risk communication on social media. One recent work used multilingual Bidirectional Encoder Representations from Transformers, a well-known transformer-based deep embedding model, and fine-tuned it by considering topical and temporal information to model topics of COVID-19 tweets [62]. On deciding topic phases via the data itself, one may use LDA and other embedding methods to model topics.

### Concluding Remarks

The current literature on the infodemic has emphasized the social media platform’s content moderation efforts [63] and fact-checking as a key risk communication strategy [64,65]. This study extends these scholarly endeavors. Predicated on an issue-attention cycle framework, we analyzed public attention on COVID-19–related topics in four Asian countries. We used a time-topic cohesive approach to automatically identify transitions in topical interests and qualitatively evaluated the topics found by local users.

Our research found that when the tweet count on COVID-19 increased, it did not lead to an increased number of topics; regardless of the tweet count, much of the public attention remained focused on a limited set of topics. The early days of the COVID-19 pandemic also involved various misinformation and hateful speech in the studied countries; fake news was one of the central topics discussed (not a peripheral topic). The proposed steps could indicate the global effects of infodemics during a pandemic and identify the emergence of misinformation and its prevalence, which will help prioritize which misinformation to debunk.

## Appendix

Multimedia Appendix 1 Data/code description, computed daily velocity/acceleration trends for each country, and derived temporal phases.

Multimedia Appendix 2 Daily topic trends on social media, the reliability of the topic modeling results, intercoder reliability, and the labeled list of major topics by country.

Multimedia Appendix 3 Daily numbers of COVID-19 confirmed cases and tweet trends by country.

## Abbreviations

GT: ground truth
LDA: latent Dirichlet allocation
RQ: research question
SARS: severe acute respiratory syndrome
