# Correction: COVID-19 Discourse on Twitter in Four Asian Countries: Case Study of Risk Communication

**DOI:** 10.2196/28926

**Published:** 2021-03-29

**Authors:** Sungkyu Park, Sungwon Han, Jeongwook Kim, Mir Majid Molaie, Hoang Dieu Vu, Karandeep Singh, Jiyoung Han, Wonjae Lee, Meeyoung Cha

**Affiliations:** 1 Data Science Group Institute for Basic Science Daejeon Republic of Korea; 2 Korea Advanced Institute of Science and Technology Daejeon Republic of Korea; 3 Electrical and Electronic Engineering Phenikaa University Hanoi Vietnam

In “COVID-19 Discourse on Twitter in Four Asian Countries: Case Study of Risk Communication” (J Med Internet Res 2021;23(3):e23272) the authors noted one error.

In the originally published article, the caption of Figure 10 was incorrectly described. It read as follows:

Daily topic trends in Vietnam based on the number of tweets.

This has been corrected to:

Daily topic trends in India based on the number of tweets.

The correction will appear in the online version of the paper on the JMIR Publications website on March 29, 2021, together with the publication of this correction notice. Because this was made after submission to PubMed, PubMed Central, and other full-text repositories, the corrected article has also been resubmitted to those repositories.

