# Supplementary material for: COVID-19 Discourse on Twitter in Four Asian Countries: Case Study of Risk Communication
Source: J Med Internet Res. 2021 Mar 16;23(3):e23272. doi: 10.2196/23272 (PMC8108572; doi:10.2196/23272)
Supplement: Multimedia Appendix 1 [file jmir_v23i3e23272_app1.pdf]

Multimedia Appendix 1

Data and Code Description

1. The Raw Dataset

We have crawled the Twitter dataset by using the existing Twint Python library and Twitter search APIs. The Twint Python library is an advanced Twitter scraping tool, written in Python. The detailed information about the scraper is explained at https://github.com/twintproject/twint. Please refer to the below code snippet to find an example usage case: https://github.com/dscig/COVID19_tweetsTopic/blob/master/code/code_tweet_collection.pdf. Please directly contact the author via shaun.park@kaist.ac.kr for detailed instruction.

Data Description

The below table is the statistics of the crawled tweets. We have set up the following keywords/hashtags by country to crawl tweets related to COVID-19. Particularly with Farsi, we have not used keywords but used hashtags, starting with “#”, mainly used among Iranians since otherwise unexpected Arabic tweets could be crawled together.

Table MA1. Statistics of the crawled tweets and the used keywords to crawl by language.

Language	Duration	Used Keyword	No. of Tweets
Korean	Jan 1 – Mar 27, 2020	- corona: 코로나<br>- wuhan pneumonia: 우한 폐렴	1,447,489
Farsi	Jan 1 – Mar 30, 2020	- corona: #کرونا<br>- coronavirus: #کروناویروس<br>- wuhan: #ووهان<br>- pneumonia: #سینه‌پهلو	459,610
Vietnamese	Jan 1 – Mar 31, 2020	- corona<br>- n-cov<br>- covid<br>- acute pneumonia: viêm phổi cấp	87,763
Hindi	Jan 1 – Mar 27, 2020	- corona: कोरोना<br>- wuhan pneumonia: वूहान निमोनिया	1,373,333

Also, below are the column names (features) and the corresponding descriptions of the dataset:

- id (type == int64): The integer representation of the unique identifier for this Tweet
- conversation_id (int64): The Tweet ID of the conversation tree's root
- created_at (datetime64): UTC time when this Tweet was created
- date (datetime64): UTC time formatted YYYY-MM-DD
- time (object): UTC time formatted h:m:s
- timezone (object): Timezone
- user_id (int64): The integer representation of the unique identifier for this User
- username (object): Screen name, handle, or alias that this user identifies themselves with
- name (object): The name of the user, as they've defined it
- place (object): Nullable When present, indicates that the tweet is associated (but not necessarily originating from) a Place
- tweet (object): The actual UTF-8 text of the status update mentions (object): Represents other Twitter users mentioned in the text of the Tweet
- urls (object): Represents URLs included in the text of a Tweet
- photos (object): Represents photo elements uploaded with the Tweet
- replies_count (int64): Number of times this Tweet has been replied to
- retweets_count (int64): Number of times this Tweet has been retweeted
- likes_count (int64): Nullable. Indicates approximately how many times this Tweet has been liked by Twitter users
- hashtags (object): Represents hashtags which have been parsed out of the Tweet text
- video (int64): The number of video elements uploaded with the Tweet
- geo (object): Nullable. Represents the geographic location of this Tweet as reported by the user or client application
- source (object): Utility used to post the Tweet, as an HTML-formatted string
- reply_to (object): Reply infos containing user_id and username

Deprecated Attributes:

- cashtags (object)
- quote_url (object)
- near (object)
- retweet (bool)
- user_rt_id (object)
- user_rt (object)
- retweet_id (object)
- retweet_date (object)
- translate (object)
- trans_src (object)
- trans_dest (object)

3. Required Basic Packages

The code has been tested running under Python 3.6.6. with the following packages installed (along with their dependencies):

- numpy == 1.16.0
- pandas == 0.23.4

4. Pre-processing Data

For the detailed tweet pre-processing and tokenizing process, please refer to the below file, including code snippet and corresponding explanation: https://github.com/dscig/COVID19_tweetsTopic/blob/master/code/code_text_preprocessing_tokenization.pdf.

[South Korea]

We have used the below Korean-specific stopwords and tokenizers.

- Text cleaning: removed special characters and URLs.
- Stopwords: find https://github.com/dscig/COVID19_tweetsTopic/blob/master/code/korean_stopwords.txt.
- Tokenizing: utilized the MeCab-Ko tokenizer (http://eunjeon.blogspot.com/).
- Please refer to the following code snippet file to find an example usage case: https://github.com/dscig/COVID19_tweetsTopic/blob/master/code/code_Korean_tokenize_sentences.pdf.

[Iran]

We have used the below Farsi-specific stopwords and tokenizers.

- Text cleaning: removed special characters and URLs.
- Stopwords: find https://github.com/dscig/COVID19_tweetsTopic/blob/master/code/farsi_stopwords.txt.
- Tokenizing: utilized the Parsivar tokenizer (https://github.com/ICTRC/Parsivar)
- Please refer to the following code snippet file to find an example usage case: https://github.com/dscig/COVID19_tweetsTopic/blob/master/code/code_Persian_tokenize_sentences.py.

[Vietnam]

We have used the below Vietnamese-specific stopwords and tokenizers.

- Text cleaning: removed special characters and URLs
- Stopwords: find https://github.com/dscig/COVID19_tweetsTopic/blob/master/code/vietnamese_stopwords.txt.
- Tokenizing: utilized the Pyvi tokenizer (https://github.com/trungtv/pyvi).
- Please refer to the following code snippet file to find an example usage case: https://github.com/dscig/COVID19_tweetsTopic/blob/master/code/code_Vietnamese_tokenize_sentences.pdf.

[India]

We have used the below Hindi-specific stopwords and tokenizers.

- Text cleaning: removed special characters, non-Hindi characters/words, and URLs.

- Stopwords: find https://github.com/dscig/COVID19_tweetsTopic/blob/master/code/hindi_stopwords.txt.

- Tokenizing: utilized word-level morphemes as tokens.

5. Decide Topical Phases

For splitting topical phases, please refer to the below code snippet: https://github.com/dscig/COVID19_tweetsTopic/blob/master/code/code_split_phases.pdf.

For deciding the phases, please refer to the below code snippet: https://github.com/dscig/COVID19_tweetsTopic/blob/master/code/code_decide_topical_phases.pdf.

6. Model Topics

We have used the Tomotopy module (https://bab2min.github.io/tomotopy/v0.6.2/en/). Please refer to the below code snippet to find an example usage case: https://github.com/dscig/COVID19_tweetsTopic/blob/master /code/code_topic_modeling.pdf.

Computed Daily *Velocity/Acceleration* Trends and Temporal Phases Derived by Country

1. South Korea

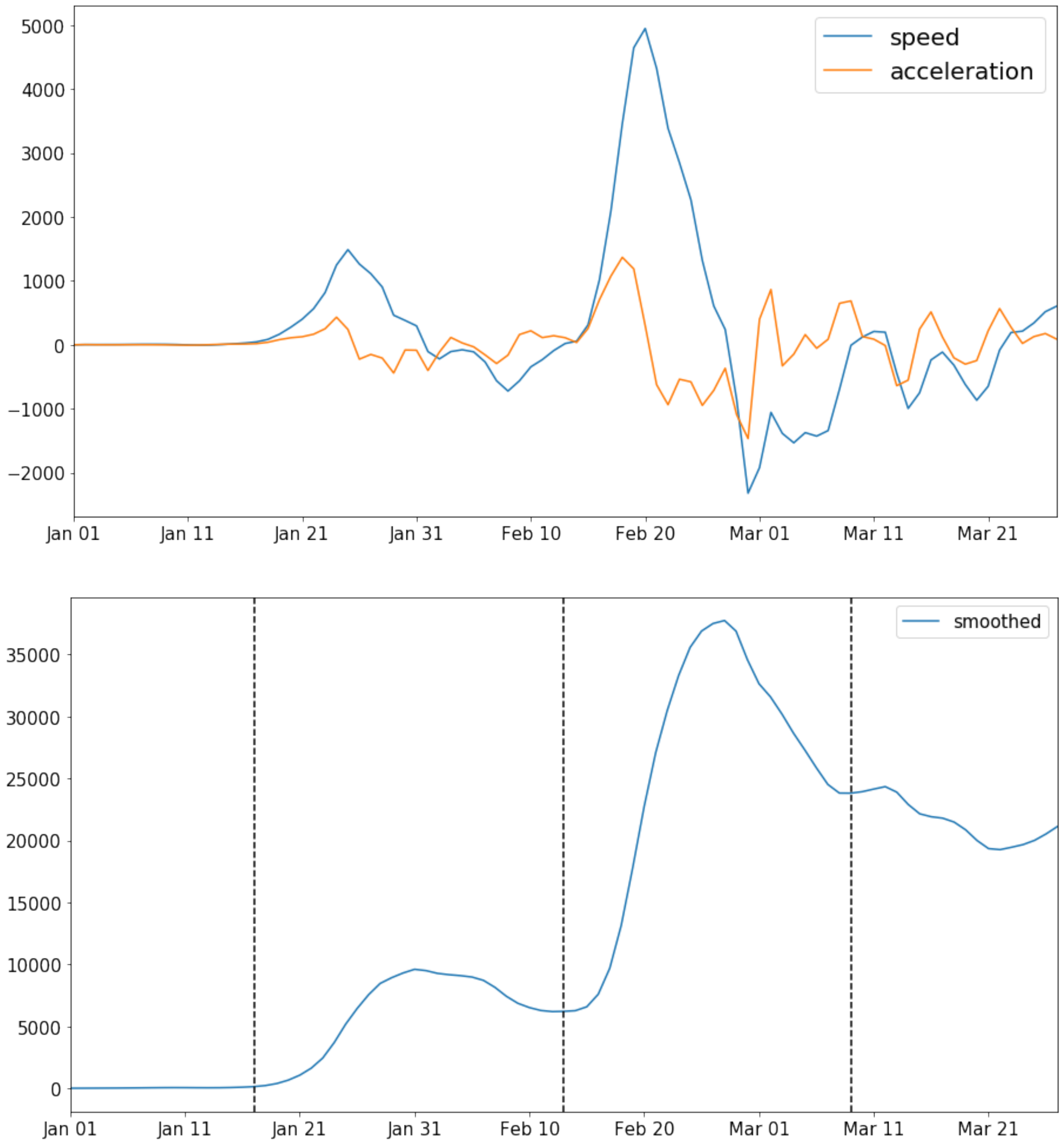

Figure MA1-1. The South Korean case: daily trends on velocity and acceleration of the # of tweets (top) and divided phases detected by vertical dash lines (bottom).

2. Iran

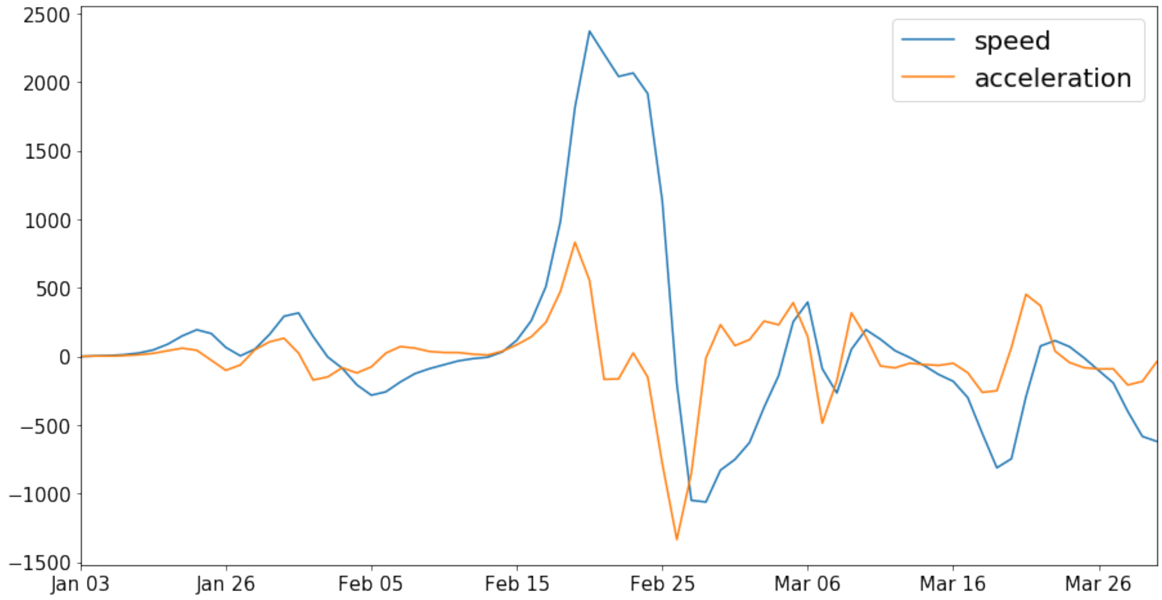

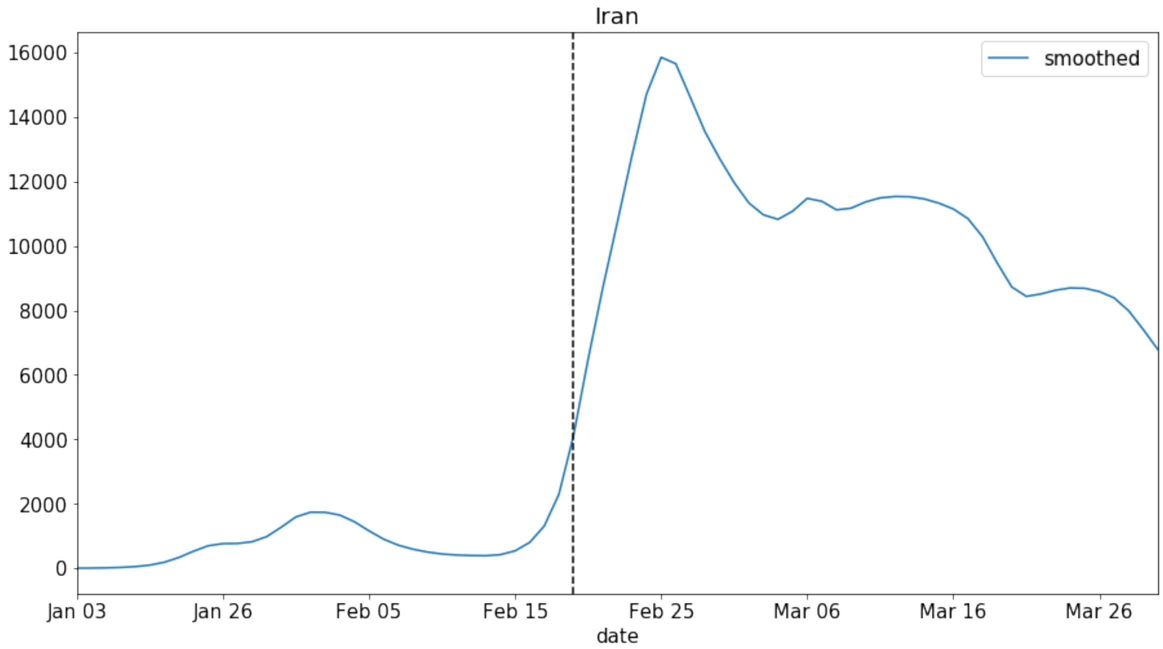

Figure MA1-2. The Iranian case: daily trends on velocity and acceleration of the # of tweets (top) and divided phases detected by vertical dash lines (bottom).

3. Vietnam

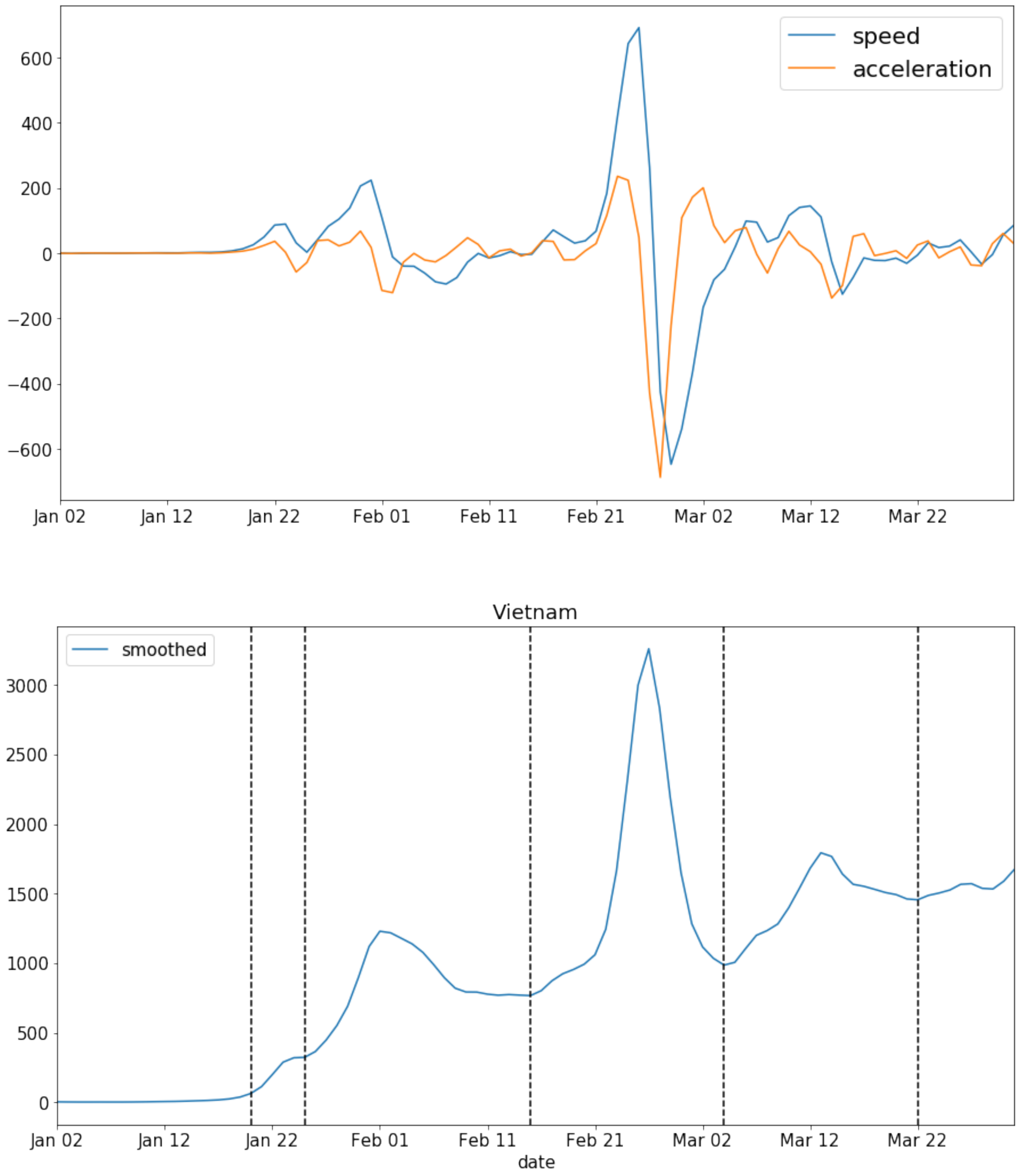

Figure MA1-3. The Vietnamese case: daily trends on velocity and acceleration of the # of tweets (top) and divided phases detected by vertical dash lines (bottom).

4. India

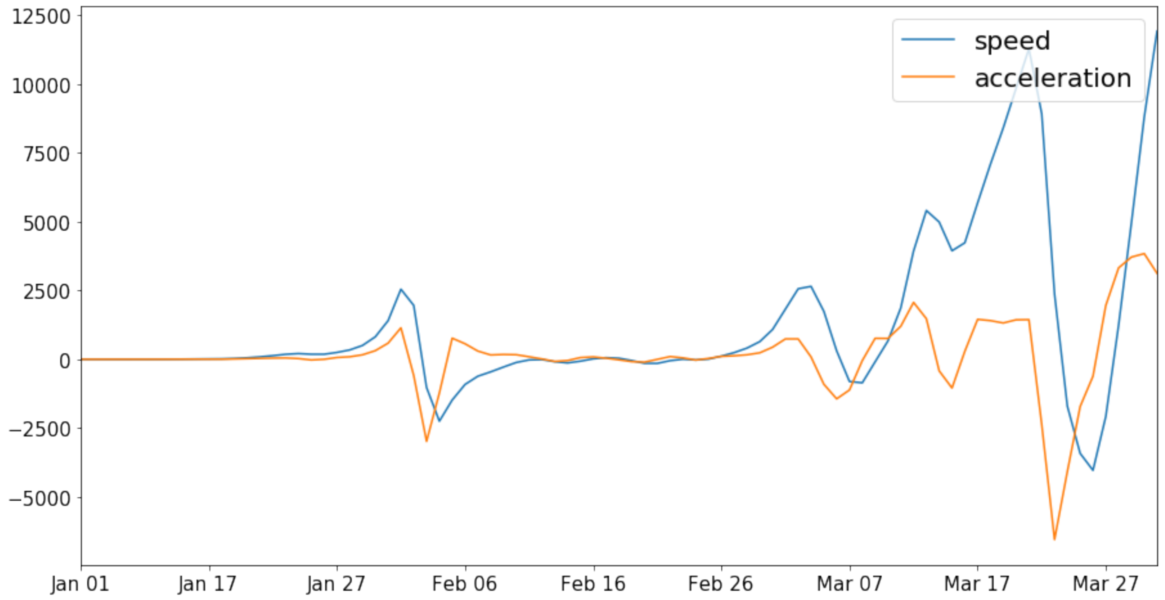

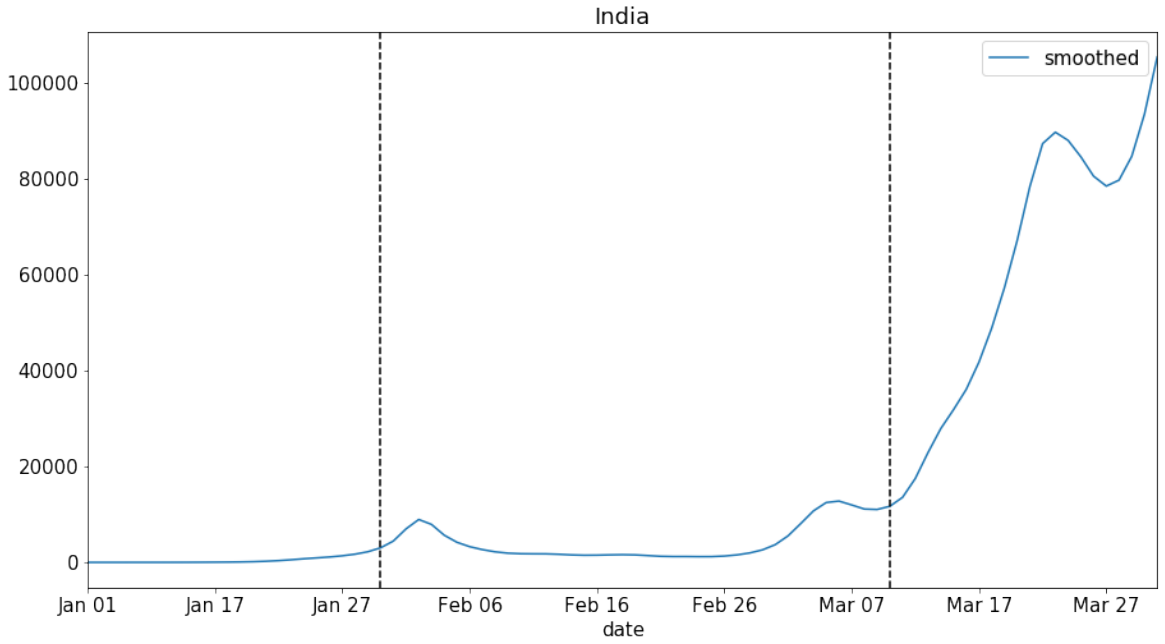

Figure MA1-4. The Indian case: daily trends on velocity and acceleration of the # of tweets (top) and divided phases detected by vertical dash lines (bottom).
