# Supplementary material for: COVID-19 Discourse on Twitter in Four Asian Countries: Case Study of Risk Communication
Source: J Med Internet Res. 2021 Mar 16;23(3):e23272. doi: 10.2196/23272 (PMC8108572; doi:10.2196/23272)
Supplement: Multimedia Appendix 2 [file jmir_v23i3e23272_app2.pdf]

Multimedia Appendix 2

The Daily Topic Trends on Social Media by Country

1. South Korea

Please refer to the "Basic Daily Trends – South Korea" subsection in the manuscript for the detailed descriptions.

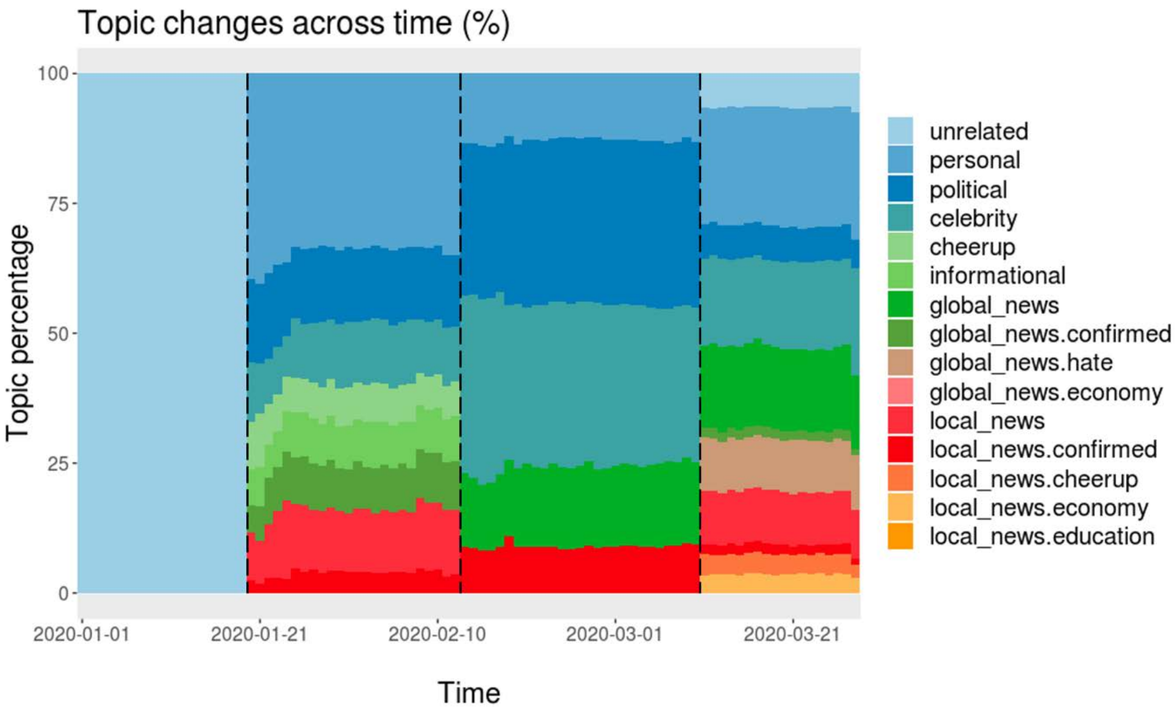

Figure MA3-1. Daily topic trends on Iran: based on % of tweets.

2. Iran

The top and middle plots in Figure MA3-2 illustrate two topic phases, their proportions, and daily topic frequencies in Farsi language tweets. Phase 0 includes global news about China and unconfirmed local news reflecting its fear of virus spread. Political issues take up a remarkable portion in this phase. At the time, the country was expecting to hold a congressional election. In phase 1, a significant increase in tweets occurs, where local news regarding the virus outbreak constitutes the majority. Notably, informational tweets about preventive measurements overshadow global news, which may be explained by disaster sociology. When people face a common disaster, naturally, more information is created and sahred that is unverified. Another reason may be due to censorship of Twitter in Iran, where Iranian citizens can access Twitter via proxies due to its blocked access (i.e., hence there may be no large-scale public campaigns). However, political tweets are still widespread because of the reasons above and public dissatisfaction about the government's response to the epidemic. This finding is also highlighted in the bottom plot of Figure MA3-2 that the U.S. is the most mentioned name after Iran and China. One possible explanation is that the outbreak puts another strain on the frail relationship between Iran and the U.S.

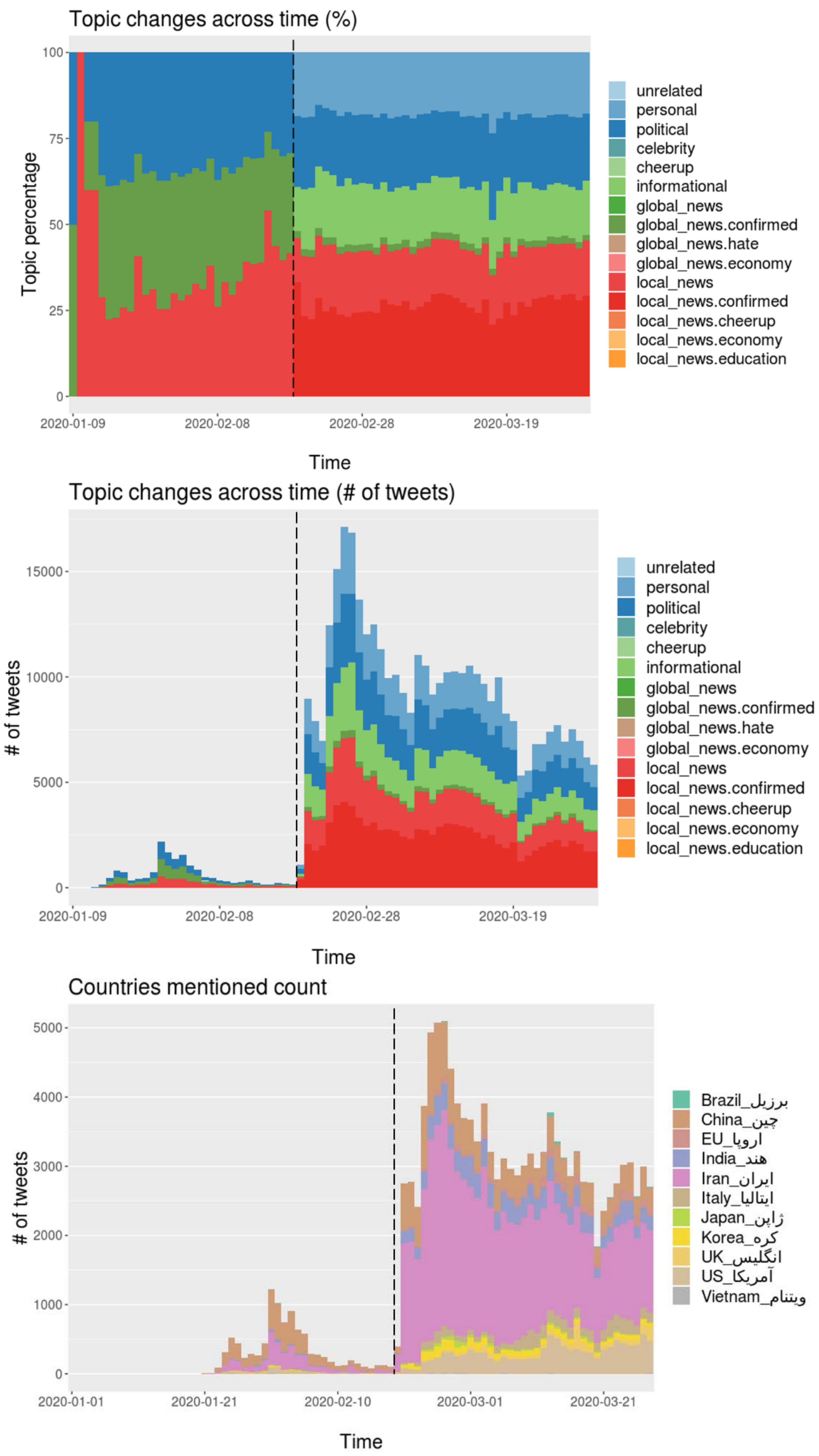

Figure MA3-2. Daily topic trends on Iran: based on % (top), based on # of tweets (mid), and based on # of tweets country names mentioned (bottom).

3. Vietnam

There are six topic phases with Vietnam, and they are visualized as in the top and middle plots in Figure MA3-3. Phase 0 is related to global news because, in this period, Vietnam did not have any confirmed patients. From phase 1 to phase 5, topics diverged, and more public attention is on the local news. Phase 3 is an exception because there had been no new confirmed cases during this time period. Similar to phase 0, the public attention goes to global news when there is no increase in local patients. Phase 3 shows increased mentions of personal topics that most did not have in other phases. It was because a conflict event that related to Korean visitors made a huge of personal tweets.

Next, we show the number of tweets that mentioned countries as in the bottom plot of Figure MA3-3. The three most mentioned countries are Vietnam, Korea, and China. Vietnam and China were mentioned frequently across phases because Vietnam is the local, and China is the original place of the pandemic. Besides, Korea was mentioned in many tweets, but they concentrated only on Phase 3. This is similar to topics changes due to the Korean visitor event in Vietnam.

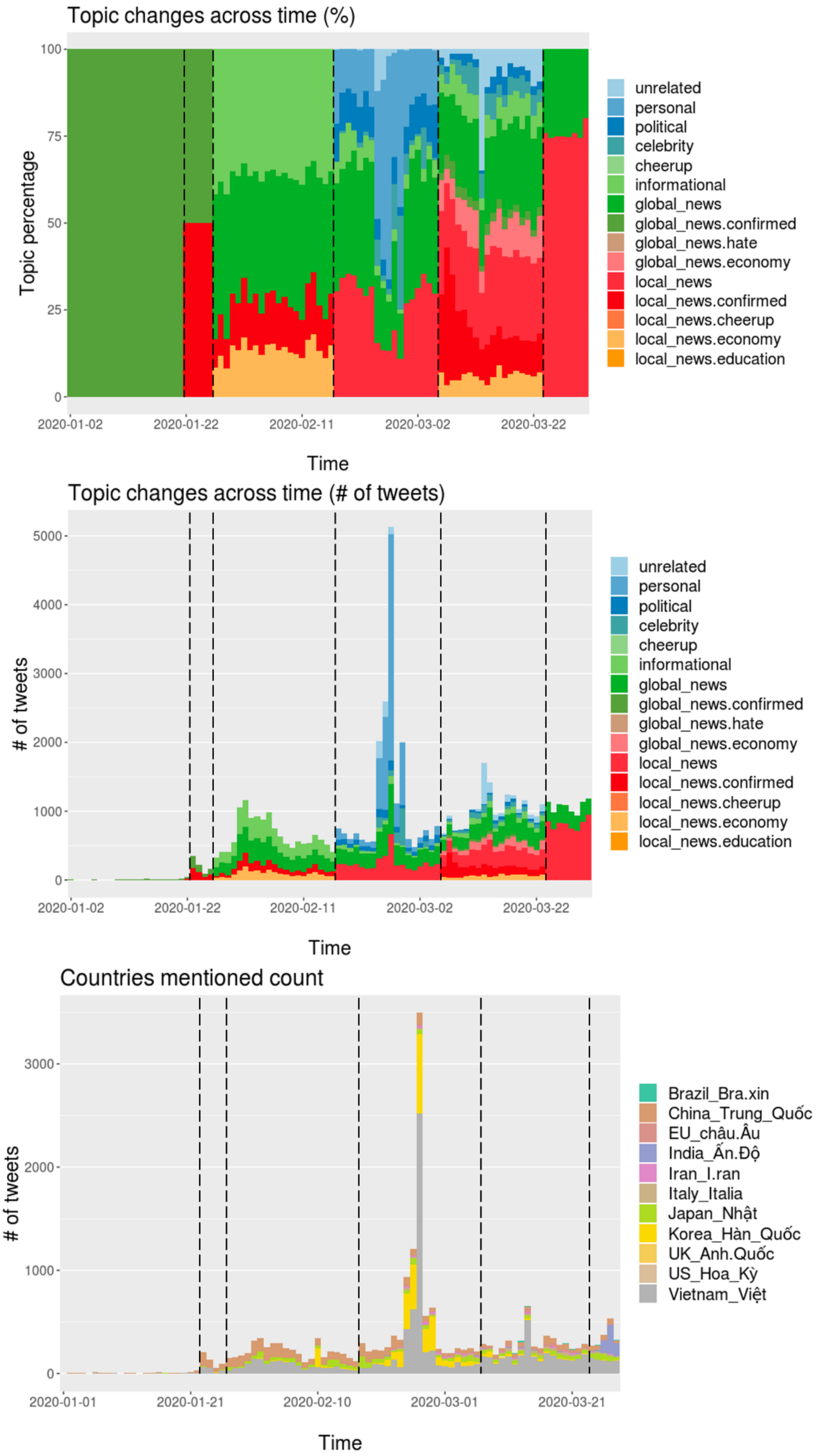

Figure MA3-3. Daily topic trends on Vietnam: based on % (top), based on # of tweets (mid), and based on # of tweets country names mentioned (bottom).

4. India

Our model identified three topic phases for Hindi-written tweets (see the top and middle plots in Figure MA3-4). Note that our study is on Hindi tweets only, and this does not include tweets generated from India that are written in English or any other language.

In India, the starting phase mainly includes tweets focused on sharing information about COVID-19 and global news about COVID-19 in China. People tended to share the news about COVID-19 and useful information on how to be safe. In phase 1, the number of topics increases. To a large degree, much of the basic information about the disease is shared during this phase, yet we also observe rumors and misinformation. The daily tweet count spikes on January 30, 2020, when the first confirmed case was announced in India. Towards the end of Phase 1 is another spike, which is led by the government announcements on new measures to block the virus (e.g., temporarily halting the visa issuing to India).

In phase 2 shows a considerable number of tweets — the proportion of informational tweets decreases, whereas local news tweets confirm new cases and increase. A marked portion of the tweets consists of hateful comments and misinformation. The hateful comments were toward particular groups of people (e.g., vegetarians, religion, etc). There are relatively few mentions directly criticizing the government.

Phase 3 witnesses an increase in mentions of country names, especially Brazil and Europe, and China and, understandably, India, as depicted in the bottom plot in Figure MA3-4. This could be attributed to a growing number of the confirmed cases in Italy, Spain, and Brazil and the news surrounding the use of Hydroxychloroquine in Brazil. The U.S. also finds considerable mentions due to the same reasons.

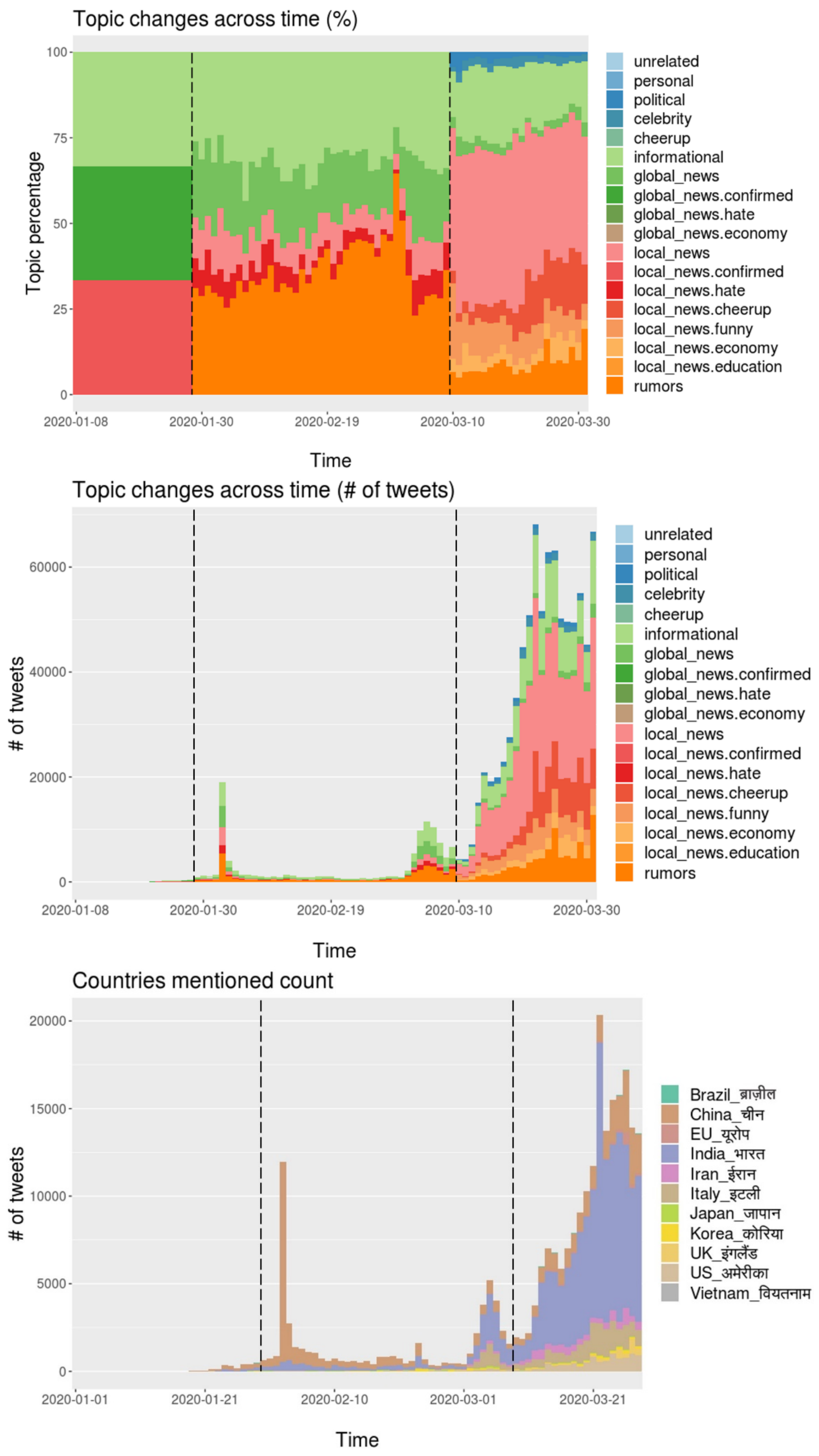

Figure MA3-4. Daily topic trends on India: based on % (top), based on # of tweets (mid), and based on # of tweets country names mentioned (bottom).

Method

[Step 3: Extract Topics – Model Topics] The Reliability of the Topic Modeling Results

Studies have utilized Twitter messages for risk communication at a different scale based on the literature survey below. Some utilize millions of messages, and to the other extreme, others utilize a thousand-scale message. For example, the research in [1] utilized LDA on 2,472 tweets, and [2] utilized 996 messages for topic modeling. Our data scale is similar; during peak times, there are far more messages that could be collected, and during low peak times, the messages are at a thousand-scale.

Regarding validation, the log-likelihood metric has been popularly used to quantify language models' quality [3]. A language model is a probability distribution over entire sentences or texts. This metric quantifies how well the model reconstructed the original document by comparing its word distribution with the original set. Perplexity (PPL), i.e., the normalized log-likelihood of a held-out test set, is an advanced language model metric. It quantifies randomness in the encoded information, similar to the entropy concept. The lower the value, the better it is at handling randomness. In this light, we can interpret low PPL to indicate better stability in reconstructing the documents since there would be a low probability of having different words when choosing the next possible token for multiple iterations.

In the case of South Korea, we reported a total of four topic phases. The number of tweets involved 507, 161K, 672K, and 366K (see Table 2) for each topic phase. The best (i.e., lowest) PPL computed for each topic phase was 23, 1296, 2120, and 2331, indicating that the model did not become particularly unstable for the first phase (i.e., when there are fewer tweets). Having much larger tweets (e.g., Phase 0 and Phase 2) also did not drastically increase the PPL value. This monotonic increase of PPL indicates that the model produces relatively stable topic model outcomes. We confirm that the same trend was observed in the four other countries.

[1] Yin J, Wang J. A Dirichlet multinomial mixture model-based approach for short text clustering. In proc. of the ACM SIG International Conference on Knowledge Discovery and Data Mining (KDD), 2014.
[2] Weng J, Lim EP, Jiang J, He Q. Twitterrank: finding topic-sensitive influential twitterers. In proc. of the ACM International Conference on Web Search and Data Mining (WSDM), 2010.
[3] Fisher D, Kozdoba M, Mannor S. Topic modeling via full dependence mixtures. In proc. of the International Conference on Machine Learning (ICML), 2020.

[Step 4: Extract Topics – Label Topics] The Intercoder Reliability of Topic Labeling

We had recruited a total of 5 annotators, two were Koreans, and the others were Iranian, Vietnamese, and Indian. All were multi-lingual and spoke fluent English. The researchers and annotators first met and decided the coding scheme based on a sample of 1,000 tweets and 30 pre-identified keywords by topic for each topic phase. The observed agreement between the two Korean annotators (i.e., accuracy) was 0.788 with the Cohen's kappa coefficient of 0.766. This task was a multi-classification with a maximum of 2 labels by topic, indicating that if one label from a multi-labeled topic was correct, only half a score (0.5) is counted. Only when both labels match, we give a full score. Given the multi-classification environment, we can say that the inter-coder reliability is reasonably good enough to proceed. The topics and keywords were discussed with the other annotators, who independently participated in the labeling task in their languages.

The Labeled List of Major (i.e., 75% Percentile) Topics by Country and the Top 10-occurring Words Therein

1. South Korea (Language: Korean)

1-1. Phase 0

Topic	Top-k words (k = 10)	labels
1	가온,진짜,루나,너무,날개,캐스,해서,나래,라푼젤,라고<br>Gaon, real, Luna, too, wings, Cass, so, Narae, Rapunzel, called	unrelated

1-2. Phase 1

Topic	Top-k words (k = 10)	labels
2	나가,바이러스,해서,진짜,근데,너무,인데,요즘,친구,만나 get out, virus, because, real, however, too, (stop-words), recently, friend, meet	personal
3	마스크,바이러스,예방,신종,착용,손소독제,미세먼지,소독,감염,손씻기<br>mask, virus, prevention, new type, wearing, hand sanitizer, micro dust, disinfection, infection, handwashing	informational
6	신종,뉴스,다음,환자,일본,확진자,감염,퇴원,국내,크루즈선<br>new type, news, Daum, patient, Japan, confirmed person, infection, discharged from hospital, domestic, cruise ship	global_news/confirmed
9	바이러스,신종,감염,가능,전염,중국,사스,전파,메르스,환자<br>virus, new type, infection, possible, transmission, China, SARS, spread, MERS, patient	informational
13	신종,정부,국민,대통령,대응,문재인,뉴스,바이러스,총리,다음<br>new type, government, citizens, president, response, Moon Jae-in, news, virus, prime minister, next	political
14	확진자,지역,동네,나왔,병원,바이러스,환자,근처,학원,저희<br>confirmed person, area, neighborhood, came out, hospital, virus, patient, nearby, private academy, us	personal
16	확진자,신종,국내,환자,발생,추가,확진,바이러스,속보,감염<br>confirmed person, new type, domestic, patient, outbreak, additional, confirmed, virus, breaking news, infection	local_news/confirmed
17	진짜,바이러스,새끼,걸리,존나,시발,마스크,남자,씨발,근데<br>real, virus, bitch, infected, damn, fucking, mask, man, fuck, however	personal
18	출처,네이버,뉴스,신종,연합뉴스,속보,확진자,경제,국내,발생<br>source, Naver, news, new type, Yonhap News, breaking news, confirmed person, economy, domestic, outbreak	local_news/confirmed,<br>global_news/confirmed
19	중국,코로나바이러스,진짜,심각,걱정,세요,무섭,한국,무서워,중국인<br>China, Coronavirus, real, serious, worry, (stop-words), scary, Korea, scared, Chinese	personal
20	진짜,취소,너무,콘서트,근데,여행,걱정,이번,인데,해서 (real, canceled, too, concert, however, travel, worry, this time, (stop-words), so	celebrity
23	감기,기침,병원,증상,독감,아프,그냥,걸리,해서,바이러스<br>cold, cough, hospital, symptoms, flu, ill, just, take it, do, virus	informational

25	바이러스,신종,취소,합니다,연기,행사,예정,입니다,드립니다,공지 virus, new type, cancel, do, postponed, event, planned, (stop-words), give, notice	local_news
29	중국,정부,한국,중국인,국민,문재인,입국,나라,북한,시진핑 China, government, Korea, Chinese, citizens, Moon Jae-in, entrance to company, country, North Korea, Xi Jinping	political
30	중국,신종,사망자,확진자,사망,출처,블로그,확진,뉴스,발생 China,new type, dead person, confirmed person, death, source, blog, confirmed, news, occurrence	global_news/confirmed
32	조심,세요,마스크,바이러스,건강,다들,요즘,해요,합니다,감기 beware, (stop-words), mask, virus, health, everybody, nowadays, let's do, I do, cold	cheerup
34	신종,대응,바이러스,확산,뉴스,지원,감염증,코로나바이러스,기자,위해 new type, response, virus, spread, news, support, infectious disease, Coronavirus, reporter, for	local_news
36	바이러스,신종,해서,나라,이번,너무,인데,상황,중국,인지 virus, new type, so, country, this time, too, (stop-words), situation, China, whether	political, personal

1-3. Phase 2

Topic	Top-k words (k = 10)	labels
3	진짜,신천지,걸리,바이러스,해서,새끼,그냥,마스크,너무,근데 real, Shincheonji, infected, virus, because, bitch, just, mask, too, however	political
6	학교,진짜,학원,확진자,나가,해서,인데,근데,연기,개학 school, real, private academy, confirmed person, go out, because, (stop-words), however, acting, starting school	personal
9	확진자,대구,확진,신천지,환자,발생,추가,뉴스,병원,검사 confirmed person, Daegu, confirmed, Shincheonji, patient, outbreak, additional, news, hospital, examination	local_news/confirmed
11	한국,일본,중국,뉴스,검사,다음,미국,바이러스,감염,확진자 Korea, Japan, China, news, examination, Daum, USA, virus, infection, confirmed person	global_news
12	진짜,잠잠,너무,빨리,만나,나가,언제,근데,해서,제발 real, calm, too, quickly, meet, go out, when, however, by doing so, please	celebrity
14	신천지,정부,대구,중국,국민,문재인,사태,나라,한다,대통령 Shincheonji, government, Daegu, China, citizens, Moon Jae-in, situation, country, do, president	political

1-4. Phase 3

Topic	Top-k words (k = 10)	labels
1	진짜,잠잠,만나,빨리,너무,언제,얼른,같이,진정,언니 real, calm, meet, soon, too, when, quickly, together, sedate, sister	celebrity
2	극복,뉴스,방역,위해,위한,기자,성금,기부,전달,지역 overcoming, news, preventive measures, in order to, for, reporter, donation, donate, delivery, area	local_news/cheerup

4	입국,여행,한국,미국,유럽,확진,해외,검사,자가격리,확진자<br>entrance to country, travel, Korea, USA, Europe, confirmed, overseas, examination, self quarantine, confirmed person	local_news, global_news
12	나라,진짜,번방,바이러스,라고,해서,한국,너무,인데,그냥<br>country, real, n-th room, virus, (stop-words), say so, Korea, too, (stop-words), just	global_news/hate
16	국민,정부,총선,문재인,대통령,나라,언론,대한민국,정치,선거<br>citizens, government, general election, Moon Jae-in, president, country, media, Korea, politics, election	political
24	조심,세요,감사,건강,합니다,해요,너무,많이,생일,행복<br>be careful, (stop-words), thank you, health, I do, let’s do, too, much, birthday, happy	unrelated
26	바이러스,한국,사태,나라,세계,상황,미국,국가,한다,정부<br>virus, Korea, event, country, world, situation, USA, nation, do, government	global_news
28	진짜,새끼,시발,존나,걸리,씨발,미친,제발,그냥,마스크<br>real, bitch, fucking, damn, infected, fuck, crazy, please, just, mask	personal
31	취소,진짜,근데,연기,올해,인데,언제,이번,상황,해서<br>canceled, real, however, postponed, this year, (stop-words),when, this time, situation, by doing so	local_news
32	지원,재난,경제,긴급,기본소득,추경,지급,위기,정부,피해<br>support, disaster, economy, emergency, basic income, supplementary budget, payment, crisis, government, damage	local_news/economy
33	한국,뉴스,다음,검사,미국,대응,나라,세계,키트,진단키트<br>Korea, news, Daum, examination, USA, response, country, world, test kit, diagnosis kit	global_news
35	해서,나가,진짜,요즘,너무,인데,근데,친구,마스크,엄마 so, go out, really, these days, too, (stop-words), however, friend, mask, mom	personal
36	진짜,콘서트,제발,빨리,너무,취소,세요,공연,눈치,무대<br>real, concert, please, quick, too, cancel, please, performance, sense, stage	celebrity
42	확진자,사망자,발생,현황,확진,사망,추가,뉴스,기준,증가<br>confirmed person, dead person, occurrence, status, confirmation, death, additional, news, standard, increase	local_news/confirmed, global_news/confirmed

2. Iran (Language: Farsi)

2-1. Phase 0

Topic	Top-k words (k = 10)	labels
1	کرونا,ویروس,کرونا,چین,ایران,کروناویروس,چینی,داره,میشه,خفاش<br>Corona, Virus, China, Iran, Coronavirus, Chinese, (stop-words), Bat	global_news/confirmed, political
2	ووهان,في,الصين,كورونا,کرونا,الصينية,مدينة,فيروس,على,إلى<br>Wuhan, (non-word), China, Corona, City, (non-words and stop-words)	local_news

2-2. Phase 1

Topic	Top-k words (k = 10)	labels
0	کرونا,في,الله,على,لا,كورونا,عن,ووهان,كل,اللهم<br>Corona, (non-words and stop-words), God, Wuhan, Total	local_news, informational
2	کرونا,نفر,ویروس,ایران,مبتلا,بیمارستان,بهداشت,آمار,قم,کروناویروس<br>Corona, Person, Virus, Iran, Infected, Hospital, Health, Statistics, Qom (city), Coronavirus	local_news/confirmed
3	کرونا,ایران,ویروس,کرونا,کروناویروس,کشور,چین,شیوع,آمریکا,دولت<br>Corona, Iran, Virus, Coronavirus, Country, China, Outbreak, The United States, Government	global_news/confirmed, political
4	کرونا,کروناویروس,کرونا,ایران,ویروس,قم,حرم,امام,خدا,الله<br>Corona, Coronavirus, Iran, Virus, Qom, Shrine, Imam, God	political, personal

3. Vietnam (Language: Vietnamese)

3-1. Phase 0

Topic	Top-k words (k = 10)	labels
15	bệnh,viêm,việt,trung_quốc,trung,phổi,corona,virus,quốc (disease, inflammation, Vietnamese, Chinese, central, lung, corona, virus, China)	global_news/confirmed

3-2. Phase 1

Topic	Top-k words (k = 10)	labels
2	nhiễm,trung_quốc,trung,quốc,việt,phát_hiện,cách_ly,vũ_hán,dương_tín h,bệnh_viện (infected, Chinese, China, Chinese, Vietnamese, detected, quarantined, Wuhan, positive, hospital)	local_news/confirmed, global_news/confirmed

3-3. Phase 2

Topic	Top-k words (k = 10)	labels
0	dịch,khẩu_trang,việt,cô_đơn,đại_dịch,trung_quốc,covid,ảnh_hưởng,bất _động_sản,thị_trường (disease, mask, Vietnamese, loneliness, pandemic, China, covid, impact, real estate, market)	local_news/confirmed, local_news/economy
1	dịch,trung_quốc,viêm,phổi,việt,trung,vũ_hán,nhiễm,dịch_bệnh,quốc (disease, China, inflammation, lung, Vietnamese, Chinese, Wuhan, infected, epidemic, Chinese)	informational, global_news
2	dịch,nghỉ,chống,học_sinh,covid,phòng,khẩu_trang,phòng_chống,phòng _dịch,trường (disease, absence from school, against, pupils, COVID, prevention, mask, prevention, epidemic prevention, school)	informational, global_news

3-4. Phase 3

Topic	Top-k words (k = 10)	labels
0	dịch,hàn_quốc,chống,việt,cách_ly,bình,trận,hợp_tác,phòng_chống,cập _nhật (epidemic, Korean, anti, Vietnamese, quarantine, stop_word, stop_word, cooperation, prevention, update)	global_news, local_news
3	bánh_mì,đất_nước,thượng_đẳng,ngon,xin_lỗi,chúng_tôi,ngáo,nổi_ting, bánh,đòi_hỏi (bread, country, elite, delicious, sorry, us, stop_word, famous, cake, demanding)	personal
4	virus,vietnam,korea,ting,news,việt,south,ngừa,trend,that (virus,Vietnam,stop-word, stop-word, stop-word, Vietnamese, stop-word, prevention, stop-word, stop-word)	personal, global_news

6	dịch,việt,quốc_gia,th_giới,hàng,ngon,du_lịch,trung_tâm,phòng_chống,chúng_tôi<br>(disease, Vietnamese, nation, world, commodity, delicious, travel, central, prevention, stop-word)	personal
8	việt,anh_chị,chăm_sóc,sức_khoẻ,cảm_ơn,video,giúp,người,repost,letuy<br>(Vietnamese, stop-word, care, health, thank, video, help, people, repost, stop-word)	personal
9	dịch,việt,cách_ly,chống,phòng,du_lịch,hàn_quốc,công_dân,nhân_viên,hàng<br>(disease, Vietnamese, quarantine, prevention, travel, Korea, citizens, employees, goods)	political
15	virus,dịch,trung_quốc,th_giới,đại_dịch,trung,kinh_t,lây_lan,có_thể,toàn_cầu<br>(virus, epidemic, china, world, pandemic, central, economic, spread, stop-word, global)	local_news, global_news
20	trend,hashtag,động,hi_vọng,chúng_mình,tốt_đẹp,trích,hàn_quốc,trend_th,concert (trend, hashtag, dynamic, hope, stop-word, nice, inject, korean, stop-word, concert)	personal
22	dịch,bác_sĩ,virus,chống,codonqua,việt,hàn_quốc,tung,ảnh_hưởng,ngân_hàng (disease, doctor, virus, anti, stop-word, vietnamese, korea, launching, influence, banking)	local_news, global_news
23	hàn_quốc,việt,idol,daegu,stan,bánh_mì,du_lịch,cách_ly,đứng,có_thể<br>(Korean, Vietnamese, idol, daegu, stop-word, bread, tourism, quarantine, stand, stop-word)	personal
24	trend,tweet,đừng,hastag,nhắc,châu,lạnh,bạn_bè,đánh_giá,hashtag<br>(trend, tweet, stop-word, hashtag, stop-word, stop-word, cold, friends, reviews, hashtag)	unrelated
25	nhiễm,virus,hàn_quốc,tử_vong,dịch,trường_hợp,nghi,việt,cập_nhật,tình_hình (infection, virus, korean, death, epidemic, case, suspect, vietnamese, update, situation)	global_news
28	việtnamtuyệtnhất,cháncác bạnthượngđẳng,du_lịch,dịch,thượng_đẳng,cách_ly,người_ta,hạ_long,việtnamquytthắngđạidịch,tham_quan<br>(stop-word,stop-word, travel, epidemic, superiority, isolation, people, Ha Long, stop-word, visit)	personal
30	việt,chúng_tôi,xin_lỗi,tôn_trọng,xem_lại,thượng_đẳng,xúc_phạm,bánh_mì,sự_thật,đất_nước<br>(Vietnamese, us, sorry, respect, review, superiority, insult, bread, truth, country)	personal
34	chúng_tôi,đất_nước,việt,cách_ly,đừng,thượng_đẳng,dịch,hàn_quốc,phát_triển,đối_xử (us, country, vietnamese, quarantine, stop-word, superiority, translation, korea, development, treatment)	personal
35	nhiễm,bệnh_nhân,việt,virus,bệnh,điều_trị,xuất_viện,bệnh_viện,trung_quốc,thuốc<br>(infection, patient, Vietnamese, virus, disease, treatment, hospital discharge, hospital, china, medicine)	informational, local_news
36	thông_báo,music,bighit,show,tip_tục,quảng_bá,sự_kiện,bùng,sân_khấu,info (announcement, music, big hit, show, resume, promotion, event, flare, stage, info)	celebrity
38	virus,dính,dịch,phòng_dịch,nhiễm,hàng,khác_thường,vắng_vẻ,khẩu_trang,hàn_quốc (virus,infection, epidemic, epidemic, infection, commodity, unusual, deserted, mask, Korea)	personal

39	tiền,army,hoàn,concert,virus,ảnh_hưởng,bighit,giúp_đỡ,đóng_góp,hỗ_trợ (Money, army, refund, concert, virus, effect, big hit, help, donation, support)	celebrity
41	chống,dịch,nghỉ,học_sinh,trưa,trường,trở_lại,phòng_dịch,phòng,sinh_viên (prevention, disease, vacation, pupils, lunch, school, back, prevention, prevention, students)	local_news
43	đẩy_lùi,việt,cách_ly,kimchi,dịch,virus,dịch_bệnh,suốt,bảo_vệ,phòng (repel, vietnamese, quarantine, stop-word, translation, virus, epidemic, transparent, protection, prevention)	global_news, local_news
45	thượng_đẳng,việt,_thức,dịch,cách_ly,quốc,thằng,đừng,con_người,anh_chị (supreme, Vietnamese, awakening, disease, quarantine, nation, guy, stop-word, people, brothers and sisters)	personal

3-5. Phase 4

Topic	Top-k words (k = 10)	labels
1	virus,việt,dịch_bệnh,tin_tức,nhiễm,news,tổng_hợp,ting,có_thể,đại_dịch (virus, Vietnamese, epidemic, news, infection, news, synthesis, stop-word, can, epidemic)	local_news, global_news
4	hoãn,dương_tính,dịch,chính_thức,cầu_thủ,bóng_đá,league,châu,giải,lo_lắng (postponement, positive, epidemic, official, player, football, league, continent, league, anxiety)	celebrity
8	đại_dịch,kinh_t,toàn_cầu,th_giới,dịch,có_thể,suy_thoái,kòkòrò,ìpínlè,pàjáwìrì (pandemic, economic, global, world, epidemic, stop-word, recession, no mean, no mean, no mean)	global_news/economy
10	chống,dịch,việt,chính_phủ,đồng,phòng_chống,cảm_ơn,ủng_hộ,cuộc_chin,tất_cả (against, epidemic, Vietnamese, government, copper, prevention, thanks, support, war, all)	local_news, global_news
11	dịch,đóng_cửa,dừng,du_lịch,hoạt_động,nhân_viên,phòng_dịch,hàng_loạt,phim,cửa_hàng (disease, shutdown, stop, travel, activity, employee, epidemic room, series, film, store)	local_news, global_news
16	dịch,cách_ly,chống,phòng,phòng_chống,dịch_bệnh,công_tác,hà_nội,tập_trung,bệnh_viện (epidemic, quarantine, prevention, prevention, epidemic, work, Hanoi, concentration, hospital)	local_news, global_news
17	bệnh_nhân,nhiễm,việt,chuyn,cách_ly,hành_khách,điều_trị,bệnh_viện,bệnh,khẩn (patient, infected, transient, transient, passenger, treatment, hospital, sick, urgent)	local_news/confirmed, global_news/confirmed
24	dịch,chịch,thời,virus,codonqua,thất_nghiệp,like,share,nuôi,follow (epidemic, fuck, time, virus, alone, unemployment, like, share, feed, follow)	unrelated
26	xô_corona,dương_tính,hoang_mang,covid_,xách,virus,th_giới,chồng,đừng,xử_l_dịch (corona, positive, confused, covid, carry, virus, world, husband, stop-word, handle)	unrelated
28	dịch,chống,phòng_chống,bảo_vệ,khẩu_trang,virus,giúp,cộng_đồng,đề_kháng,vệ_sinh (disease, prevention, prevention, protection, masks, viruses, help, community, resistance, hygiene)	informational
29	dịch,đóng_cửa,việt,châu,phong_tỏa,lây_lan,nhập_cảnh,biên_giới,news,canada (epidemic, closure, vietnamese, continental, blockade, spread, entry, border, news, Canada)	political

33	nhiễm,việt,ghi_nhận,bệnh_nhân,công_bố,trường_hợp,bệnh,tổng_số,dương_tính,nâng (infection, transient, record, patient, statement, case, illness, total, positive, increase)	local_news/confirmed
37	dịch,ảnh_hưởng,doanh_nghiệp,tiền,thị_trường,có_thể,thời,đại_dịch,kinh_t,hỗ_trợ (disease, impact, business, money, market, can, era, pandemic, economic, support)	global_news/economy, local_news/economy
38	xét_nghiệm,âm_tính,nhiễm,kt_quả,dương_tính,tip_xúc,bệnh_nhân,tình_hình,nhân_viên,trump (test, negative, infection, result, positive, exposure, patient, situation, employee, Trump)	local_news, global_news
41	virus,đại_dịch,trung_quốc,dịch,th_giới,mượn,diệt,sống,chúng_ta,nhạc (virus, pandemic, China, translation, world, borrow, destroy, live, us, music)	local_news, global_news
42	nhiễm,bệnh_nhân,hà_nội,cách_ly,dương_tính,liên_quan,tip_viên,bạch,tip_xúc,trúc (infection, patient, hanoi, isolated, positive, related, flight attendant, no mean, contact, no mean)	local_news/confirmed
43	virus,nhiễm,việt,trung_quốc,lây_nhiễm,chống,pháp,dính,hàn_quốc,news (virus, infection, vietnamese, China, infection, anti, french, infection, Korean, news)	local_news, global_news
44	dịch,theo_dõi,ứng_dụng,nhiễm,nghi,phun,cách_ly,khử_trùng,phòng_dịch,dịch_vụ (disease, monitoring, application, infection, suspicion, spray, isolation, disinfection, epidemic prevention, service)	local_news
45	thuốc,virus,chữa,điều_trị,vaccine,thử_nghiệm,chống,ngừa,bệnh,sốt_rét (medicine, virus, cure, treatment, vaccine, testing, prevention, prevention, disease, malaria)	global_news

3-6. Phase 5

Topic	Top-k words (k = 10)	labels
5	dịch,đại_dịch,ảnh_hưởng,thời,doanh_nghiệp,bất_động_sản,hỗ_trợ,có_thể,tiền,thị_trường (disease, pandemic, impact, fashion, business, real estate, assistance, can, money, market)	local_news
9	nhiễm,bệnh_nhân,việt,bệnh_viện,bệnh,cách_ly,bạch,xét_nghiệm,hà_nội,dương_tính (Infection, patient, Vietnamese, hospital, illness, quarantine, transparency, test, positive, positive)	local_news, global_news
12	dịch,virus,chống,đại_dịch,th_giới,news,cập_nhật,nhiễm,trung_quốc,hàng (disease, virus, anti, pandemic, world, news, updates, infected, China, goods)	local_news, global_news
14	dịch,chống,phòng_chống,phòng,cách_ly,việt,đồng,thủ_tướng,ủng_hộ,dịch_bệnh (epidemic, prevention, prevention, quarantine, Vietnamese, bronze, prime minister, advocacy, epidemic)	local_news

4. India (Language: Hindi)

4-1. Phase 0

Topic	Top-k words (k = 10)	labels
2	कोरोना,वायरस,संदिग्ध,चीन,भर्ती,अस्पताल,मरीज,भारत,सामने,बिहार<br>Corona, Virus, Suspected, China, Admitted, Hospital, Patient, India, As opposed (This word might have other meanings based on context), Bihar (Bihar is a state in India)	global_news/confirmed, local_news/confirmed, informational

4-2. Phase 1

Topic	Top-k words (k = 10)	labels
2	कोरोना,वायरस,बंद,स्कूल,खाने,मार्च,दिल्ली,चिकन,मांस,आदेश<br>Corona, virus, close, school, to eat, march (procession), Delhi, meat (and chicken), order	local_news, rumors
4	होली,होगी,कोरोना,वायरस,होगा,गाय,गोबर,वातावरण,शुद्ध,कंडे<br>Holi (is a festival in India),ST, corona, virus, ST, cow dung, environment, pure, eggs	local_news, global_news
5	कोरोना,वायरस,बचाव,क्या,बचने,उपाय,सावधानी,रहें,लक्षण,कैसे<br>Corona, virus, prevent, ST, preventive, way, precaution, ST, symptoms, ST	informational
6	वायरस,परमात्मा,खाना,मांस,हम,क्या,आदेश,चलते,अन्य,भयंकर<br>Virus, The Divine, food (it could a noun or a verb), meat, ST, ST, order, to walk (depends on context), others, severe	rumors
8	होली,गोबर,कोरोना,गाय,वायरस,कंडे,जलाने,नष्ट,रंगों,पलाश<br>Holi, cow dung, corona, cow, virus, prickles, to burn, destroy, colors, pliers (not sure though)	local_news, rumors, informational
9	कोरोना,वायरस,चीन,भारत,सरकार,दुनिया,चपेट,अमेरिका,क्या,रोक<br>Corona, virus, China, India, government, world, grip, USA, ST, to ban/stop.	global_news
12	कोरोना,वाइरस,वजह,ग़लत,वोट,फैल,वायरस,चाइना,दिल्ली,गोश्त<br>Corona, virus, reason, wrong, vote, spread, virus, China, Delhi, meat	informational
14	कोरोना,वायरस,पीएम,नमस्ते,मोदी,आदत,मीडिया,झेल,बचें,हमें<br>Corona, virus, P.M. (prime minister), Namaste, Modi, habit, media, fraud/trick, to be careful, ST	rumors
15	बंद,मांस,खाना,चीन,वरना,वायरस,पड़ेगा,कोरोना,जबरदस्ती,साधना<br>stop/ban, meat, food, China, otherwise, virus, ST, corona, forcefully/forced, meditation	rumors
19	कोरोना,हाथ,नमस्कार,करवा,नमस्ते,वायरस,शाकाहारी,संस्कृति,सनातन,भारतीय<br>Corona, hand (in context of someone’s involvement in something), Namaskar (greeting, salutation), doing (context - who is supporting this), Namaste, virus, vegetarian, culture, Sanatan (Sanatan dharma - eternal duties incumbent upon people in Hinduism), Indian	informational, rumors
20	कोरोना,वायरस,मरीज,संदिग्ध,अस्पताल,जांच,दिल्ली,भारत,लोगों,गए<br>Corona , virus, patient (medical context), suspected, hospital, tests, Delhi, India, people, ST	informational, local_news

21	कोरोना,बहिष्कार,स्वदेशी,वायरस,मलेरिया,व्हायरस,विदेशी,नाही,मरें,अपनायें,<br>Corona, boycott, indigenous, virus, malaria, viruses, foreign, ST, die, adopt	informational, global_news, local_news
26	कोरोना,देश,जाये,सालों,गद्दारों,जय,नही,ठीक,भड़काऊ,जाए<br>Corona, country, go, (its an offensive word), traitors, to hail, ST, correct, provocative, ST	local_news/hate
30	कोरोना,चीन,भारत,पाकिस्तान,रोना,सोना,टन,वायरस,कर्ज,पड़ोसी<br>Corona, China, India, Pakistan, cry, gold, ton, virus, debt, neighbor	local_news/hate, global_news
31	गंदगी,मनुष्य,खाते,पशु,मांस,रहते,पक्षी,खाने,वायरस,फैला<br>Garbage, humans, eat, animals, meat, ST, birds, eat, viruses, spread	informational
34	परमात्मा,आदेश,आपदाएं,वायरस,कोरोना,चीन,जैसी,प्रकार,उल्लंघन,आया<br>The Divine, order, disasters, virus, corona, China, ST, type, violation, ST	rumors
36	कोरोना,वायरस,हाथ,दूरी,मुँह,नाक,उससे,बार,साबुन,बुखार<br>Corona, virus, hand, distance, mouth, nose, ST, ST, soap, fever	informational, rumors
42	जी,कोरोना,योगी,बना,हज,जनता,हाउस,अस्पताल,बेड,अपील<br>Yes, corona, yogi (most probably in context of Chief Minister of Uttar Pradesh state in India), ST, Haj (holy pilgrimage of Muslims), public, house, hospital, bed, appeal	local_news
44	कोरोना,वायरस,अल्लाह,चीन,अब,क्या,चपेट,नमाज़,नमाज,लगा<br>Corona, virus, Allah (Muslims call The Divine as Allah), China, now, what, grip, Namaz (Muslims' prayer), Namaz (Muslims' prayer), felt	global_news, rumors
45	भगवान,इलाज,जाने,आगे,बचने,आ,साइंस,कितनी,लो,संविधान<br>The Divine, cure, ST, next/forward, escape, ST, science, how much, ST, constitution	rumors
47	होली,कोरोना,वायरस,रंग,हाथ,बार,नमस्ते,वैदिक,पलाश,डरने<br>Holi, corona, virus, color, hand, this time, Namaste, Vedic, pliers, to fear	informational
49	वायरस,कोरोना,भारत,खतरा,वापस,जान,चीन,यहां,देखकर,मेरी<br>Virus, corona, India, threat, back, life (most probably in context of fearing for his life) , China, ST, seeing, ST	local_news, global_news, rumors

4-3. Phase 2

Topic	Top-k words (k = 10)	labels
0	कोरोना,वायरस,भारत,टेस्ट,किट,क्या,दवा,सरकार,इलाज,तैयार<br>Corona, virus, India, test, kit, ST, medicine, government, cure, ready	informational, rumors, local_news/cheerup
1	कोरोना,वायरस,बंद,मार्च,स्कूल,चलते,सरकार,स्थगित,आदेश,रद्द<br>Corona, virus, closed, march, school, moving, government, postponed, order, canceled	informational, local_news
8	कोरोना,सरकार,वायरस,आर्थिक,पैकेज,मोदी,करोड़,लाख,राहत,रही<br>Corona, government, virus, financial, package, Modi, crore (1 crore = 10 million), lakh (1 lakh = 100,000), relief	local_news/economy
9	कोरोना,लोग,मर,गरीब,भूख,क्या,देश,सरकार,नही,मजदूर<br>Corona, people, dead, poor, hungry, what, country, government, no, laborers	local_news, local_news/economy
10	कोरोना,वायरस,बचाव,लेकर,जी,संक्रमण,श्री,जिला,बैठक,मुख्यमंत्री<br>Corona, virus, rescue, ST, ST, infection, ST, district, meeting, Chief	local_news

	Minister	
12	कोरोना,रहें,वायरस,सुरक्षित,पालन,हम,बचाव,सरकार,देश,सावधानी<br>Corona, stay, virus, safe, obey, ST, rescue, government, country, caution	informational
13	कोरोना,करोड़,लाख,दान,लड़ने,देश,रुपये,राहत,मदद,कोष<br>Corona, crore, lakh, donation, fight, country, rupee (INR currency), relief, help, fund	local_news/cheerup
14	कोरोना,जी,सरकार,जाए,लोग,वायरस,नही,चाहिए,देश,निवेदन<br>Corona, ST, government, go, people, virus, ST, want, country, request	local_news, informational
15	कोरोना,देश,हम,जय,भारत,जी,हारेगा,जीतेगा,सब,श्री<br>Corona, country, we, to hail, India, ST, will lose, will win, ST, ST	local_news/cheerup
17	कोरोना,सरकार,देश,दिल्ली,रही,केजरीवाल,क्या,लोगों,लोग,भारत<br>Corona, government, country, Delhi, ST, Kejriwal (Arvind Kejriwal, Chief Minister of Delhi), ST, people, people, India	local_news, rumors
22	कोरोना,सरकार,कांग्रेस,राहुल,गांधी,वायरस,जी,गायब,देश,मोदी<br>Corona, government, congress, Rahul, Gandhi, (Rahul Gandhi is leader of the main opposition party in India) virus, law, missing, country, Modi	local_news, political
24	कोरोना,वायरस,मौत,संख्या,भारत,देश,संक्रमित,लोगों,अब,मामले<br>Corona, virus, death, number, India, country, infected, people, now, case	local_news, global_news
27	कोरोना,विश्व,संस्कृति,महामारी,आज,प्रार्थना,नववर्ष,वायरस,संकल्प,माँ<br>Corona, world, culture, pandemic, today, prayer, new year, virus, resolve, mother	rumors
36	कोरोना,वायरस,मीडिया,गो,खतरनाक,इलाज,मूत्र,अब,सोशल,न्यूज़<br>Corona, virus, media, go, dangerous, cure, (cow) urine, nowST social, news	informational
37	कोरोना,पॉजिटिव,वायरस,कनिका,मरीज,कपूर,अस्पताल,संदिग्ध,जांच,रिपोर्ट<br>Corona, positive, virus, Kanika (Kanika Kapoor is an India celebrity) , patient, camphor, hospital, suspected, investigation, report	celebrity, local_news
38	कोरोना,जनता,कर्फ्यू,बजे,प्रधानमंत्री,जी,मार्च,देश,मोदी,वायरस<br>Corona, public, curfew, a time unit, Prime minister, ST, march, country, Modi, virus	local_news
39	कोरोना,नही,क्या,देश,वायरस,लोग,अब,वो,मोदी,पता<br>Corona, ST, ST, country, virus, people, now, ST, Modi, to know (could have other contextual meanings)	local_news
40	कोरोना,वायरस,मास्क,लोग,रही,पुलिस,नही,लोगों,डाउन,दे<br>Corona, virus, mask, people, ST, police, ST, people, down, give	informational, local_news
41	कोरोना,जिहाद,देश,वायरस,मुस्लिम,भारत,पाकिस्तान,अब,लोग,बम<br>Corona, jihad (means holy war, but most probably it is used here in some derogatory context), country, virus, Muslim, India, Pakistan, ST, people, bomb(s)	rumors
45	कोरोना,वायरस,अब,क्या,करो,आज,आ,सब,नही,दिन<br>Corona, virus, now, what, do, today, come, all, no, day	local_news/funny
