# Supplementary material for: COVID-19 Discourse on Twitter in Four Asian Countries: Case Study of Risk Communication
Source: J Med Internet Res. 2021 Mar 16;23(3):e23272. doi: 10.2196/23272 (PMC8108572; doi:10.2196/23272)
Supplement: Multimedia Appendix 3 [file jmir_v23i3e23272_app3.pdf]

Multimedia Appendix 3

Daily Numbers of COVID-19 Confirmed Cases and Tweet Count Trends by Country

1. South Korea

The first confirmed case was identified on January 20, 2020 [1]. From early January till January 20, the daily numbers of tweets were relatively small, whereas the number sharply increased on January 25, as depicted in Figure 3. January 25 was the date when the Korean government increased the travel warning level on Wuhan city and Hubei province to suggest to evacuate from there, and this sign may affect the communication on Twitter. On February 18, the number sharply increased that had not been shown before, and it may be due to the 31st confirmed case related to a cult religious group in Daegu city. After the 31st confirmed case has been found, the quarantine authority tried rigorous testing focusing on Daegu, and the number of the confirmed cases was drastically increasing until mid-March. The tweet trends also follow the same pattern. However, the official epidemic phases announced by the government, divided by the vertical dash lines in the figure, seem to lag from the increasing number of tweets. Hence, we could say that the epidemic phases may not explain the online communication trends in Korea well enough.

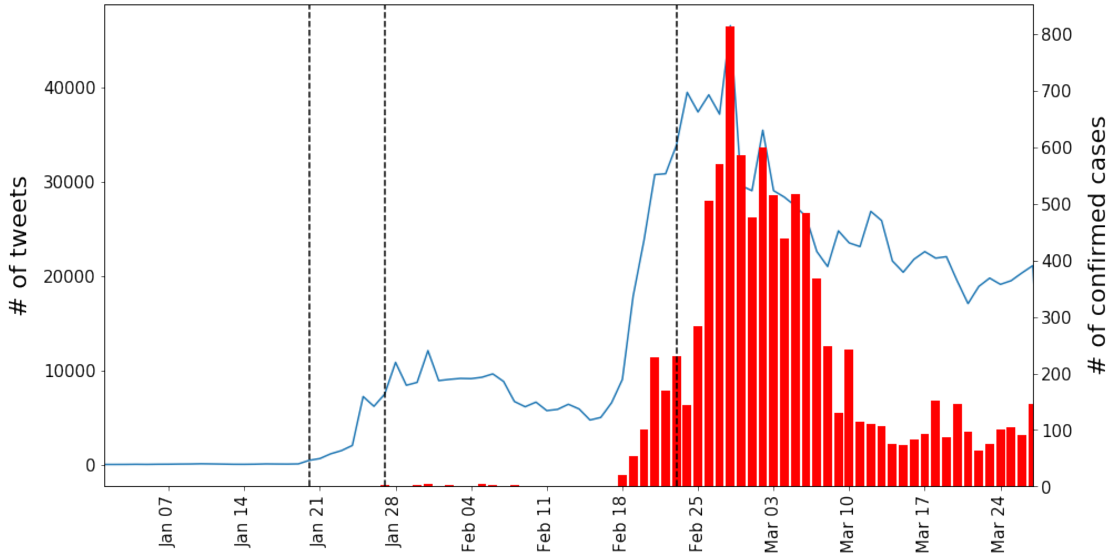

Figure MA2-1. Daily trends on South Korea: start/end dates of the official epidemic phases (vertical dash lines), trends of # of tweets (blue lines), and that of # of the confirmed cases (red bars).

2. Iran

On February 19, two people tested positive for SARS-CoV-2 in the city of Qom [2]. After this date, we see a significant surge in the number of tweets, and it reaches a peak in a few days (i.e., a peak shown on February 25). On February 23, the government changed the alert from white to yellow. Although the number of confirmed cases keeps increasing, the number of tweets starts to decrease gradually with a little fluctuation, as shown in Figure 4. Therefore, the trends of these two numbers show different patterns in contrast to Korean tweets. Meanwhile, the government gradually increased preventive measures, and several cities with the highest rate of infection were announced hot spots or red zones. Overall, they did not place the whole country under the red alert. However, the government announced new guidance and banned all trips on March 25. On March 28, the president said that 20% of the country's annual budget would be allocated to fight the virus, which might be implicitly a sign of the red alert.

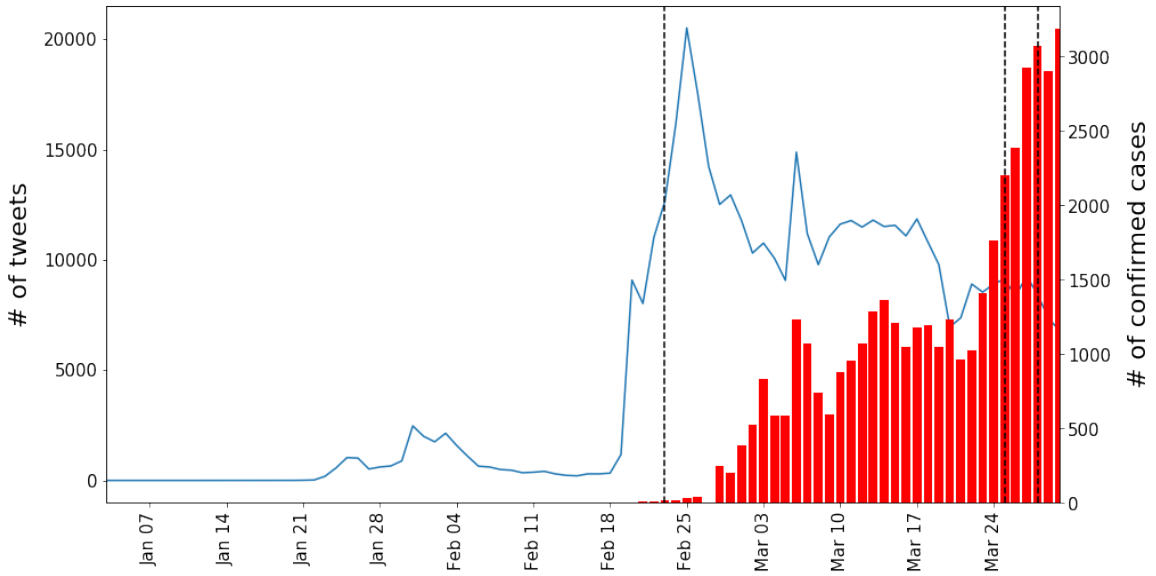

Figure MA2-2. Daily trends on Iran: start/end dates of the official epidemic phases (vertical dash lines), trends of # of tweets (blue lines), and that of # of the confirmed cases (red bars).

3. Vietnam

On January 23, 2020, Vietnam officially confirmed the first two COVID-19 patients, who come from Wuhan, China [3]. After that, the number of tweets increased sharply and reached to peak in early February, as shown in Figure 5. Although a few new cases were detected, the number of tweets tended to decrease and remained stable. In the second half of February, there are no new cases. However, the number of tweets increased rapidly and created a new peak. This peak could not remain for a long time. Two possible reasons can explain this trend. The first is that the pandemic has spread over the world. The second is that the last cases in Vietnam were treated successfully. After a long time with no new cases, Vietnam had confirmed continuously new cases in Hanoi and many other cities from March 6. The number of tweets of this phase increased again and remain stable at a relatively higher level than the initial phase.

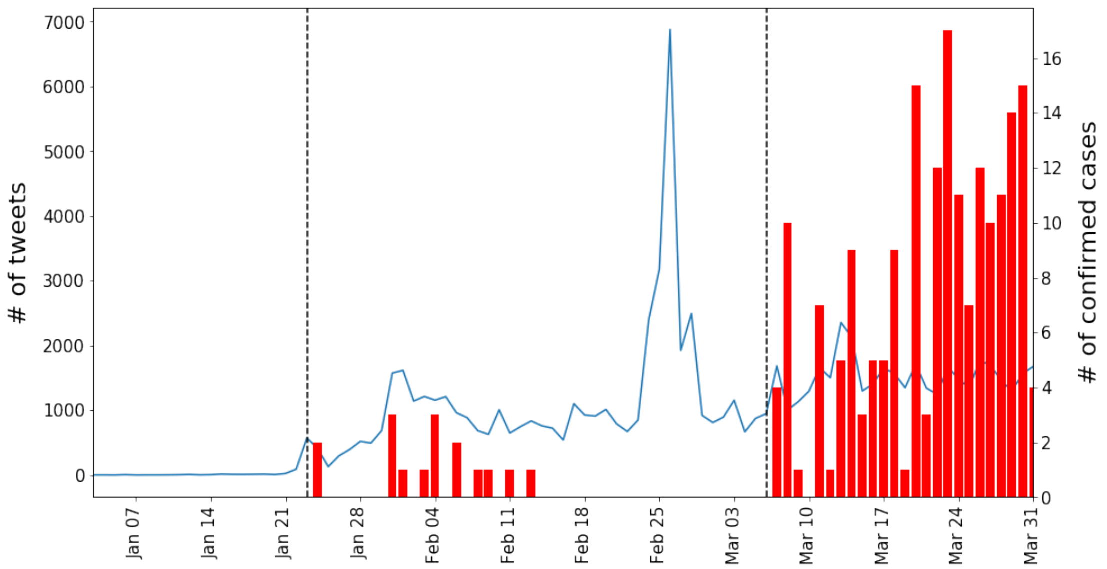

Figure MA2-3. Daily trends on Vietnam: start/end dates of the official epidemic phases (vertical dash lines), trends of # of tweets (blue lines), and that of # of the confirmed cases (red bars).

4. India

The first case of COVID-19 was confirmed on January 30, 2020 [4]. The number of cases quickly rose to three on account of students returning from Wuhan, China. Throughout February, no new cases were reported, and the first weeks of March also saw a relatively low number of cases. The number of cases, however, picked up numbers from the fourth week of March, notable were the 14 confirmed cases of Italian tourists in the Rajasthan province. This eventually led to the government of India declaring a complete lock-down of the country. The daily number of tweets followed a similar trend as that of the number of cases as depicted in Figure 6. First confirmed cases around January 30, 2020, caused a sudden spike in the number of tweets, that subsided in February. First COVID-19 fatality on March 12 and some other COVID-19 local events led to an exponential increase in tweets. The tweets peaked on March 22 when the government declared lock-down of areas with infected cases and started trending downwards after that. It is strange that the government's declaration of nationwide lock-down on March 24 only caused a small spike in the number of tweets and trend continued downwards. However, March 31 saw a significant spike in the number of tweets owing to confirmation of mass infections in a religious gathering. Overall, the tweet trends seem to be synonymous with the government's release of official information (e.g., # confirmed cases and fatalities on COVID-19).

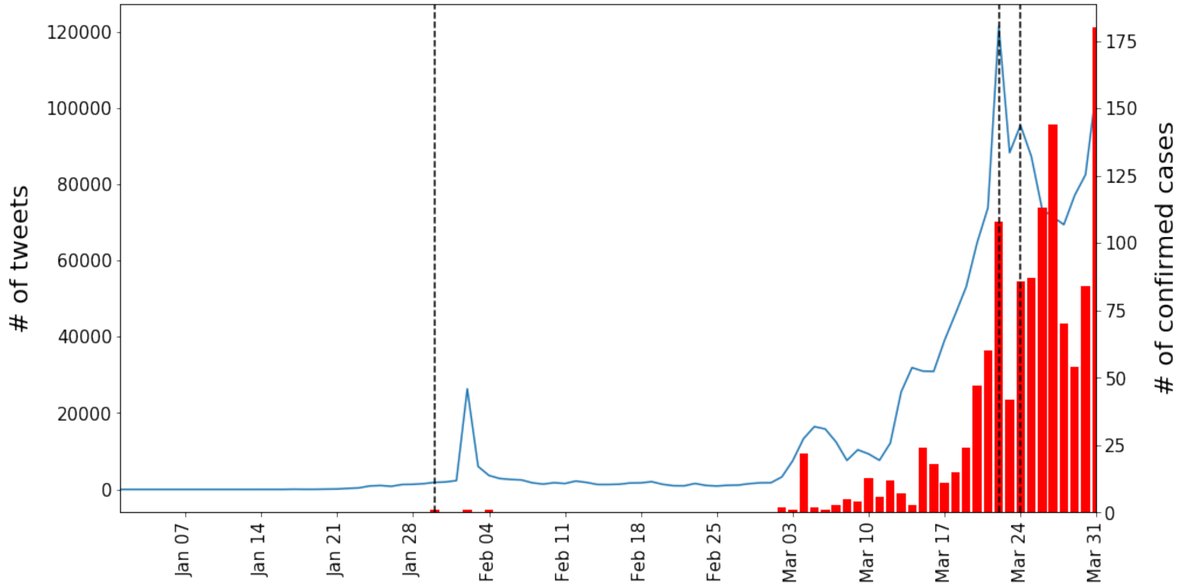

Figure MA2-4. Daily trends on India: start/end dates of the official epidemic phases (vertical dash lines), trends of # of tweets (blue lines), and that of # of the confirmed cases (red bars).

References

1. COVID-19 pandemic in Korea. Wikipedia 2020. URL: https://bit.ly/3fy4SZp.
2. COVID-19 pandemic in Iran. Wikipedia 2020. URL: https://bit.ly/3ftQDV5.
3. COVID-19 pandemic in Vietnam. Wikipedia 2020. URL: https://bit.ly/35BOyC2.
4. COVID-19 pandemic in India. Wikipedia 2020. URL: https://bit.ly/37wIdsN.
